# Genomic Insight Into the Predominance of Candidate Phylum Atribacteria JS1 Lineage in Marine Sediments

**DOI:** 10.3389/fmicb.2018.02909

**Published:** 2018-11-29

**Authors:** Yung Mi Lee, Kyuin Hwang, Jae Il Lee, Mincheol Kim, Chung Yeon Hwang, Hyun-Ju Noh, Hakkyum Choi, Hong Kum Lee, Jongsik Chun, Soon Gyu Hong, Seung Chul Shin

**Affiliations:** ^1^Division of Polar Life Sciences, Korea Polar Research Institute, Incheon, South Korea; ^2^Department of Polar Science, University of Science and Technology, Daejeon, South Korea; ^3^Division of Polar Paleoenvironment, Korea Polar Research Institute, Incheon, South Korea; ^4^Division of Polar Earth-System Sciences, Korea Polar Research Institute, Incheon, South Korea; ^5^School of Biological Sciences, College of Natural Sciences, Seoul National University, Seoul, South Korea; ^6^Unit of Polar Genomics, Korea Polar Research Institute, Incheon, South Korea

**Keywords:** candidate division, Atribacteria, JS1, single-cell genomics, Antarctica, Ross Sea, marine sediment

## Abstract

Candidate phylum Atribacteria JS1 lineage is one of the predominant bacterial groups in anoxic subseafloor sediments, especially in organic-rich or gas hydrate-containing sediments. However, due to the lack of axenic culture representatives, metabolic potential and biogeochemical roles of this phylum have remained elusive. Here, we examined the microbial communities of marine sediments of the Ross Sea, Antarctica, and found candidate phylum Atribacteria JS1 lineage was the most abundant candidate phylum accounting for 9.8–40.8% of the bacterial communities with a single dominant operational taxonomic unit (OTU). To elucidate the metabolic potential and ecological function of this species, we applied a single-cell genomic approach and obtained 18 single-cell amplified genomes presumably from a single species that was consistent with the dominant OTU throughout the sediments. The composite genome constructed by co-assembly showed the highest genome completeness among available Atribacteria JS1 genomes. Metabolic reconstruction suggested fermentative potential using various substrates and syntrophic acetate oxidation coupled with hydrogen or formate scavenging methanogens. This metabolic potential supports the predominance of Atribacteria JS1 in anoxic environments expanding our knowledge of the ecological function of this uncultivated group.

## Introduction

The subseafloor biosphere, which contains huge amounts of microbial biomass, plays a significant role in biogeochemical cycling and the remineralization of organic materials (Kallmeyer et al., 2012; Parkes et al., 2014). Enormous progress has been made in studies of subseafloor microbes. As a first step toward understanding ecological functions of the subseafloor, many studies have evaluated microbial community structures from a variety of seas and their relationships with physicochemical parameters (Kato et al., 1997; Inagaki et al., 2006; Webster et al., 2006a; Schauer et al., 2009; Zinger et al., 2011; Hamdan et al., 2012; Jorgensen et al., 2012; Parkes et al., 2014; Carr et al., 2015; Corinaldesi, 2015; Nunoura et al., 2016). These surveys revealed that the microbial composition is shaped by the physicochemical properties of the environment, including oxygen concentration and nutrient availability (Zinger et al., 2011; Jorgensen et al., 2012; Parkes et al., 2014). The majority of microorganisms belonged to unidentified and uncultured phyla, making it difficult to infer their metabolic activities and ecological functions (Handelsman, 2004; Webster et al., 2004; Inagaki et al., 2006; Orcutt et al., 2011; Rinke et al., 2013; Macaulay and Voet, 2014; Corinaldesi, 2015; Eloe-Fadrosh et al., 2016; Nobu et al., 2016). Thus, understanding the metabolism and functions of candidate divisions in the subseafloor biosphere is a major challenge and will provide substantial insights into the roles of microbes in global biogeochemical cycles.

*Candidatus* (*Ca*.) Atribacteria is a candidate phylum that was proposed (Nobu et al., 2016) to include members of the OP9 (Hugenholtz et al., 1998) and JS1 (Rochelle et al., 1994; Webster et al., 2004) lineages. The JS1 lineage is a predominant bacterial group in subseafloor sediments, especially in some strictly anoxic organic-rich environments or gas hydrate-containing sediments, and methanogenic meromictic lakes (Webster et al., 2004, 2006a; Inagaki et al., 2006; Orcutt et al., 2011; Gies et al., 2014; Parkes et al., 2014; Carr et al., 2015; Ruff et al., 2015; Nobu et al., 2016; Nunoura et al., 2016; Saxton et al., 2016). To elucidate the physiology of the JS1 lineage, culture-independent approaches such as isotope enrichment studies and genomics have been applied (Webster et al., 2006b, 2011; Dodsworth et al., 2013; Lloyd et al., 2013; Rinke et al., 2013; Carr et al., 2015; Nobu et al., 2015, 2016). Stable isotope probing and enrichment studies revealed the incorporation of acetate and glucose into members of JS1, suggesting heterotrophic metabolism (Webster et al., 2006b, 2011). Single-cell amplified genome (SAG) analysis or metagenomic approaches to marine sediments from Aarhus Bay, Etoliko Lagoon, and the Adélie Basin, biofilms from a terephthalate-degrading reactor, and monimolimnion of the meromictic Sakinaw Lake also indicated that members of JS1 are heterotrophic anaerobes that lack respiratory capacity (Dodsworth et al., 2013; Lloyd et al., 2013; Rinke et al., 2013; Carr et al., 2015; Nobu et al., 2016). The high-coverage JS1 genomes recovered from meromictic Sakinaw Lake and the mesophilic bioreactor revealed the capacity to catabolize organic acids such as propionate and acetate via the methylmalonyl-CoA pathway and lack of sugar fermentation pathways (Nobu et al., 2016). However, the low-coverage JS1 genomes from marine sediments (<22%) did not allow detailed description of metabolic potential in spite of the predominance of JS1 in those environments and, thus, the metabolism and ecological functions of the JS1 lineage in marine sediments remain largely unknown (Lloyd et al., 2013; Rinke et al., 2013; Carr et al., 2015; Nobu et al., 2015, 2016).

In this study, we investigated the vertical profiles of bacterial and archaeal communities along a 3.96-m-long sediment core in the Ross Sea, Antarctica, using 454 pyrosequencing of 16S rRNA genes and found that the JS1 lineage was the predominant bacterial group throughout the sediments below the surface. Given the dearth of genomic information on the JS1 lineage from marine sediments, we analyzed single-cell genomes of the JS1 lineage to understand the metabolic potential and assess their ecological role in the environment.

## Materials and Methods

### Sampling and Handling

Sediment samples were collected from a 3.96-m gravity core (DG12-GC06) retrieved from the western Ross Sea, Antarctica (75°39.5684′ S, 165°23.8382′ E, water depth 859 m), on January 15, 2012, on the research vessel (RV) ARAON. The entire round core was cut in half, and samples were collected with a sterile (autoclaved) spatula at 20-cm intervals, including the surface (Lee, 2017). Samples for molecular biological analysis were stored untreated at -80°C and those for recovery of single cells were suspended in 20% glycerol (v/v) and stored at -80°C until analysis. Water content, grain size distribution, total nitrogen content and total organic carbon content of samples were analyzed. The content of particles larger than sand-size was determined by wet sieving, and the content of silt- and clay-sized particles was analyzed using a Micrometric Sedigraph 5120. Contents of total nitrogen (TN) and total carbon (TC) were analyzed using a Flash EA 1112 element analyzer and the analytical precisions are within ±0.02% for TN and ±0.1% for TC. Total organic carbon (TOC) content was determined by subtracting total inorganic carbon from total carbon content. Total inorganic carbon content was analyzed for a 2N HCl-dissolved portion using a UIC 5030 coulometer.

### Genomic DNA Extraction and Amplification

Genomic DNA was extracted from approximately 0.5 g of sample using a FastDNA Spin Kit for Soil (Qbiogene, Carlsbad, CA, United States). The 16S rRNA gene was amplified by polymerase chain reaction (PCR) using primers 27F (Lane, 1991)/518R (Kato et al., 1997) and 8F (3′-CTCAGAGTAGTCCGGTTGATCCYGCCGG-5′)/519R (3′-ACAGAGACGAGGTDTTACCGCGGCKGCTG-5′) with barcodes for bacterial and archaeal community analysis, respectively. PCR was carried out with 30 μL reaction mixtures containing 2× Dream Tag Green PCR master mix (Thermo Fisher Scientific, Waltham, MA, United States), 1 μM each primer, and approximately 10 ng of genomic DNA. The PCR procedure included an initial denaturation step at 94°C for 3 min, 30 cycles of amplification (94°C for 1 min, 55°C for 1 min, and 72°C for 1.5 min), and a final extension step at 72°C for 5 min. Each sample was amplified in triplicate and pooled. PCR products were purified using a LaboPass purification kit (Cosmogenetech, Seoul, South Korea). Because of PCR failure for samples below 120 cm below the sea floor (cmbsf) with archaeal primers, these samples were not included in further analyses. For the bacterial community in the 140 and 320 cmbsf samples, barcodes were overlapped and thus these samples were excluded from further analysis.

### Analysis of Microbial Community

Sequencing of amplicons was carried out by Chun Lab (Seoul, South Korea) using a 454 GS FLX sequencer (Roche, Branford, CT, United States). Preprocessing was conducted using PyroTrimmer (Oh et al., 2012). Sequences were processed to remove primer, linker, and barcode sequences. The 3′-ends of sequences with low quality values were trimmed when average quality scores for a 5-bp window size were <20. Sequences with ambiguous nucleotides or <200 bp were discarded. Sequence clustering was performed by CLUSTOM (Hwang et al., 2013) with a 97% similarity cutoff. Chimeric reads were detected and discarded using the *de novo* chimera detection algorithm of UCHIME (Edgar et al., 2011). Taxonomic assignment for representative sequences of the bacterial and archaeal OTUs was conducted using an EzTaxon-e database search (Kim et al., 2012). Prefix “B_” for bacterial OTUs and “A_” for archaeal OTUs were attached. The habitats of archaeal sequences were inferred by matching OTU representative sequences to those in the GenBank database using a BLAST search with ≥97% sequence similarity cutoff.

### Statistical Analysis

The relative similarities of bacterial and archaeal communities among samples were calculated by Bray-Curtis similarity using an OTU abundance matrix prepared by logarithmic transformation of percent abundance + 1 by PRIMER v6 (Clarke and Gorley, 2006). Spearman correlations between the major JS1 OTU, designated B_OTU1, and major archaeal OTUs (≥3% in relative abundance) were performed using the R package to find significant relationships.

### Single-Cell Sorting, Genome Amplification, Sequencing, and Phylogenetic Analysis

Based on bacterial community results, a sample from 40 cmbsf which harbored 39.6% JS1 was selected for single-cell sorting. Samples preserved in 20% glycerol at -80°C were centrifuged for 1 min at 9,300 ×*g* and 0.5 mL of the supernatant was mixed with 100 μL of 100× TE buffer (pH 8.0) and 5 mL of filtered and autoclaved seawater, packed in dry ice, and sent to Bigelow Lab (East Boothbay, ME, United States). Physical isolation of single cells was performed by fluorescent-activated cell sorting in a 384-well plate. After single-cell sorting, lysis of single cells and amplification of the single-cell genome by multiple displacement amplification (MDA) were performed. MDA product subsamples were used as a template in PCR for amplification of bacterial 16S ribosomal RNA genes using the primer sets 27F and 1492R (Lane, 1991). Eighteen SAGs belonging to the JS1 lineage were sequenced using a MiSeq sequencer system (Illumina) at Chun Lab. For phylogenetic analysis of JS1 lineage, 16S rRNA gene sequences from this study were aligned with those of JS1 retrieved from the SAGs and metagenomic data sets (Table 1) and two dominant OTUs of the JS1 lineage, B_OTU1 and B_OTU3, obtained from pyrosequencing results (Supplementary File 1) using jPhydit (Jeon et al., 2005). A phylogenetic tree was constructed using maximum-likelihood method based on the general time reversible model (Felsenstein, 1981; Nei and Kumar, 2000) with the gamma distribution with invariant sites using MEGA 6 (Tamura et al., 2013). The robustness of the tree topologies was assessed by bootstrap analyses based on 1,000 replications.

**Table 1:** Genomic characteristics of Atribacteria JS1 lineages.

Genome ID	NCBI accession	Habitat	Location	RAST ID by Nobu et al^1^	RAST ID in this study^2^	Genome size (Mbases)	Genome completeness (%)^3^	Genome completeness (contamination) (%)^4^	Gene count in functional categories	Reference
RS JS1-cSAG	NCRO00000000	Marine sediment	Ross Sea, Antarctica		379707	2.3	90	85.5 (1.7)	831	This study
SCGC AD-561-N23		Marine sediment	Adelie Basin, Antarctica		1476961.3	0.2		5.2 (0)	32	Carr et al., 2015
SL SAG co-assembly	AWNT00000000	Meromictic lake water	Sakinaw lake, Canada	54176	138344	2.09	81	63.8 (0)	805	Rinke et al., 2013
SCGC AAA252-M02	AQYX00000000	Meromictic lake water	Sakinaw lake, Canada		190053	2.2		65.5 (0)	816	Rinke et al., 2013
SL MG bin		Meromictic lake water	Sakinaw lake, Canada	54179	193095	0.34	31	18.2 (0.3)	155	Nobu et al., 2016
SCGC AB-164-G04	AQRY00000000	Meromictic lake water	Sakinaw lake, Canada	54177	193096	0.33	23	29.3 (0)	135	Rinke et al., 2013
SCGC AAA255-E04	ASLT00000000	Meromictic lake water	Sakinaw lake, Canada	100175	193097	0.15		8.6 (0)	49	Rinke et al., 2013
SCGC AAA255-G05	ASPA00000000	Meromictic lake water	Sakinaw lake, Canada	100178	138345	1.65		56.3 (1.7)	608	Rinke et al., 2013
SCGC AAA255-N14	ASPC00000000	Meromictic lake water	Sakinaw lake, Canada	100183	193098	1.42		58.5 (0)	482	Rinke et al., 2013
SCGC AB-164-A22	AQSW00000000	Meromictic lake water	Sakinaw lake, Canada	100187	193099	0.94		29.3 (0)	389	Rinke et al., 2013
Aarhus Bay SAG I22	CDPM01000000	Marine sediment	Aarhus Bay, Baltic Sea, Denmark	94538	161940	1.04	22	22.4 (0)	300	Lloyd et al., 2013
Aarhus Bay SAG B17	CDPL01000000	Marine sediment	Aarhus Bay, Baltic Sea, Denmark	54178	193103	1.13	7	7.8 (0)	221	Lloyd et al., 2013
JGI 0000014-F07	ASOZ00000000	Marine sediment	Etoliko Lagoon, Greece	54175	193102	0.32	8	8.6 (0)	92	Rinke et al., 2013
JGI 0000079-L04	ASOY00000000	Terephthalate degrading bioreactor		54174	193100	0.95	25	35.3 (0)	434	Rinke et al., 2013
TA biofilm MG bin		Terephthalate degrading bioreactor		54181	193101	2.01	86	52.2 (5.2)	688	Nobu et al., 2016
JGI 0000059-I14	ASLS00000000	Terephthalate degrading bioreactor		54173	193104	0.49	33	17.2 (0)	173	Rinke et al., 2013

*^1^All assemblies are publicly available at RAST (http://rast.nmpdr.org/) by logging in with username and password “guest.” All RAST IDs start with “666666” (e.g., 6666666.54176). ^2^Re-annotated assemblies for the comparison of RS JS1-cSAG and other Atribacteria JS1 lineages in RAST and data are publicly available on RAST. All RAST IDs with the exception of SCGC AD-561-N23 start with “666666” (e.g., 6666666.379707). ^3^Genome completeness was estimated using the Conserved Single Copy Gene set as previously described by Rinke et al. (2013). ^4^Genome completeness and contamination were estimated by CheckM.*

### Genome Assembly and Annotation

Quality trimming and assemblies of sequencing reads were performed using CLC Genomics Workbench (version 8.0). Quality trimming was performed with the parameters “limit = 0.05” and “maximum number of ambiguities = 2.” For assembly, the bubble size and word size was set to 50 and 22, respectively. A read mapping was performed after the initial contig creation and contig regions with no reads map were removed. Average nucleotide identity (ANI) values of 18 SAGs were calculated to identify whether the SAGs were identical to each other using the dnadiff script of the MUMmer tool (version 3.0) (Kurtz et al., 2004). To construct the composite SAG (cSAG), designated RS JS1-cSAG, reads from 18 SAGs were pooled and assembled. Assembled contigs were annotated with Rapid Annotation using Subsystem Technology (RAST) (Aziz et al., 2008). Additional enzyme commission (EC) numbers for coding sequences were obtained from the KEGG Automatic Annotation Server (KAAS) (Moriya et al., 2007).

### Genome Completeness Estimation

Genome completeness was estimated using the Conserved Single Copy Gene (CSCG) set (bacteria: 139 markers), as previously described by Rinke et al. (2013). To identify CSCGs in the RS JS1-cSAG, protein sequences for PFAM matches were analyzed (Finn et al., 2016). The search for protein families using HMMER3 was performed using an online tool^1^. Proteins with the resulting best hits above precalculated HMM cutoffs were selected as CSCGs, and the completeness was estimated as the ratio of CSCGs detected to total CSCGs. Since the CSCGs were defined to occur only once in at least 90% of all genomes (*n* = 1,516), the number of total CSCGs was normalized to 90% (Rinke et al., 2013). CheckM was also used for estimation of genome completeness and contamination using their domain-specific markers (bacteria: 104 markers) (Parks et al., 2015).

### Nucleotide Sequence Accession Numbers

Sequences obtained by pyrosequencing technology have been deposited in the Short Read Archive of the National Center for Biotechnology Information under accession numbers 6660657–6660675 under the BioProject number PRJNA380995. RS JS1-cSAG sequences have been deposited in the Whole-Genome Shotgun project at DDBJ/EMBL/GenBank under accession number NCRO00000000, and the annotated assemblies for the RS JS1-cSAG (Genome ID 6666666.379707) are available at RAST^2^ by logging in with a guest account (username and password = “guest”). The 16S rRNA sequences were submitted to NCBI GenBank under accession numbers KY888007–KY888024.

## Results

### Environmental Factors of Sediments

The amount of TN, TOC, and water in the core varied according to the lithologic characteristics of the sediment (Supplementary Figure S1). The core can be divided into three units: the upper unit of greenish-gray diatomaceous mud with minor ice-rafted debris (core depth: 0–120 cmbsf); the middle unit of dark greenish-gray to dark gray diamicton and sandy mud (120–360 cmbsf); and the lower unit of light greenish-gray diatomaceous mud (360–396 cmbsf). Clay- and silt-sized grains were dominant throughout the core, but the content of coarser-sized grains (sands and gravels) reached up to 20% in the middle unit. Water content of the upper unit sediment (63–64 wt.%) was much higher than that of the middle and lower unit sediments (∼30 wt.%) (Supplementary Figure S1), which can be attributed to differences in grain size distribution and depositional setting. TOC content was generally low (<1 wt.% throughout the core) (Supplementary Figure S1). Contents of TOC and TN were higher in the upper unit than in the lower units (Supplementary Figure S1), which may be related to higher primary production during postglacial periods than glacial periods.

### Depth Profile of Microbial Phyla and Major OTUs

**FIGURE 1:**
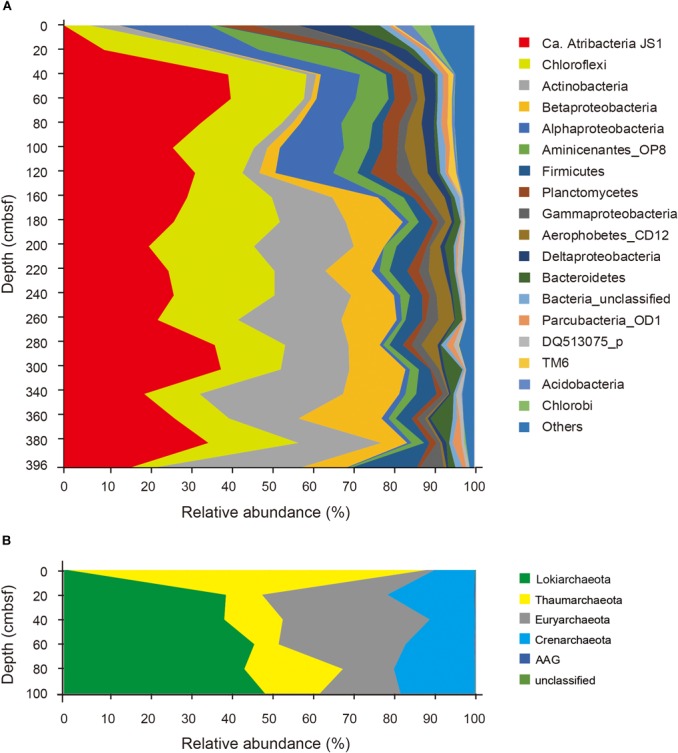
Relative abundance of sequenced bacterial **(A)** and archaeal communities **(B)** at the phylum level or the class level in the case of Proteobacteria along the depth of the core.

**FIGURE 2:**
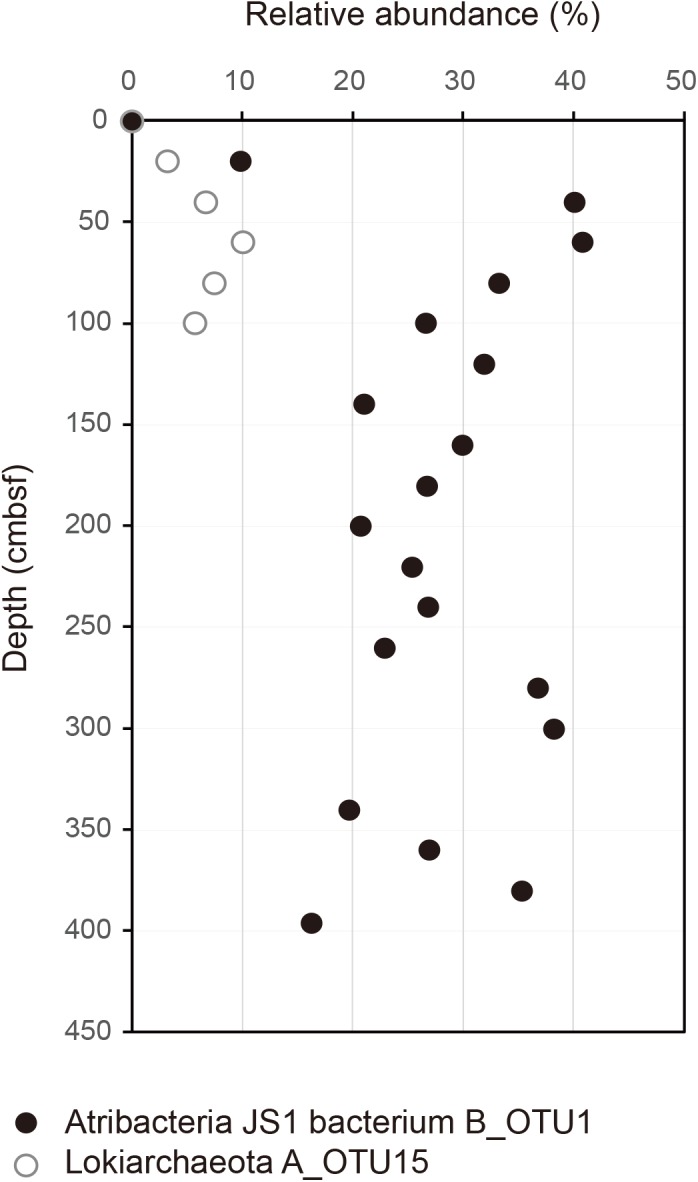
Relative abundance of B_OTU1 of JS1 and archaeal OTU, A_OTU15 along the depth of the core. B_OTU1 is the most predominant OTU of JS1 and A_OTU15 is an archaeal OTU with a significant correlation with B_OTU1. Closed circles indicate JS1 OTU, B_OTU1, and open circles indicate archaeal OTU A_OTU15 of Lokiarchaeota.

Relative abundances of the sequenced bacterial phyla showed stratification along the depth of the core (Figure 1A) and bacterial community composition was clustered into three groups: 0 cmbsf, 20–120 cmbsf, and 160–393 cmbsf (Supplementary Figure S2A). In surface sediment, Alphaproteobacteria (21.9%), Deltaproteobacteria (12.1%), Gammaproteobacteria (11.0%), Planctomycetes (8.6%), Bacteroidetes (7.7%), Actinobacteria (7.0%), Chlorobi (4.7%), and Acidobacteria (4.2%) were more abundant, whereas *Ca.* Atribacteria JS1 (9.8–40.8%), Chloroflexi (6.0–26.0%), Actinobacteria (1.9–35.6%), Betaproteobacteria (0.5–20.1%) and Aminicenantes (1.0–19.5%) were found in higher abundance at and below 20 cmbsf. *Ca.* Atribacteria JS1 and Chloroflexi dominated throughout the subsurface sediments, accounting for 22.2 to 59.2% of bacterial communities. Notably, a single OTU, B_OTU1, accounted for a high proportion of the sequenced JS1 lineage (9.1–40.1%) at and below 20 cmbsf (Figure 2). Relative abundance of Aminicenantes was higher from 20 to 160 cmbsf, while the relative abundance of Actinobacteria and Betaproteobacteria was distinctively higher at and below 160 cmbsf than in the upper sediments (Figure 1A).

Archaeal phyla detected throughout the sediments included Lokiarchaeota (formerly Marine Benthic Group B), Thaumarchaeota, Euryarchaeota, Crenarchaeota, and ancient archaeal group (AAG) (Figure 1B). Like the bacterial communities, archaeal communities were clearly divided at 20 cmbsf (Supplementary Figure S2B). In the surface sediment, Thaumarchaeota was the most dominant archaeal phylum, accounting for 87% of the sequenced archaeal community. However, in sediments at and below 20 cmbsf, candidate division Lokiarchaeota was the most abundant group, accounting for 39.1–48.9% of the sequenced archaeal community, followed by Euryarchaeota (12.3–35.9%) and Crenarchaeota (10.9–21.2%). Major archaeal OTUs with proportions of >3% in each sample also clearly changed along the depth (Supplementary Figure S3). Four OTUs of Thaumarchaeota accounted for 86.5% of the archaeal community at the surface, whereas their abundance at and below 20 cmbsf ranged from 5.9 to 23.5% (Supplementary Figure S3). Notably, two OTUs of Lokiarchaeota, A_OTU2 and A_OTU15, with proportions of 1.0 and 0.0%, respectively, at 0 cmbsf increased to 38.8 and 10%, respectively, in deeper sediments. The Euryarchaeota, OTU A_OTU5, which was rarely recovered at 0 cmbsf, accounted for 25.5% at 40 cmbsf.

To investigate the co-occurrence of the single dominant OTU of the JS1 lineage (B_OTU1) and major archaeal OTUs (≥3% relative abundance), Spearman correlation coefficients were calculated. B_OTU1 of JS1 was positively correlated with A_OTU15 of Lokiarchaeota (*r* = 0.94, *p* < 0.01) (Supplementary Table S1 and Figure 2). The low 16S rRNA gene similarity of the archaeal OTUs that showed significant correlations did not allow them to be assigned to any known taxa (Supplementary Figure S3). To infer the ecophysiological properties of archaeal OTU, A_OTU15, which had a significant positive correlation with major JS1 OTU (B_OTU1), the habitats of sequences with >97% similarity with A_OTU15 were recovered and analyzed. Most of the sequences recovered were from marine sediments, and particularly from gas-hydrate bearing sediments, cold or hydrocarbon seeps, or mud volcanoes (Supplementary Table S2).

### Genome Characteristics of Single Amplified Genomes and Phylogenetic Diversity of JS1 Lineage

**FIGURE 3:**
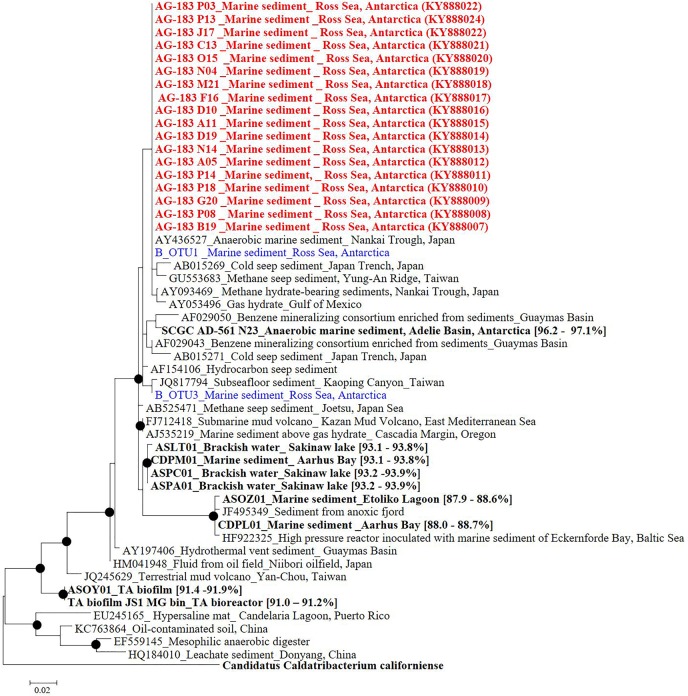
Phylogenetic tree based on 16S rRNA gene sequences from *Candidatus* Atribacteria JS1 lineage. Red colored sequences represent sequences retrieved from single-cell genomes obtained in this study while blue colored sequences were obtained by pyrosequencing of 16S rRNA amplicons in this study. B_OTU1 and B_OTU3 were the most abundant Ca. Atribacteria JS1 sequences. Sequences from other studies are indicated with GenBank accession ID numbers, the habitats, and location of the organisms from which the sequences were obtained. Bold sequences were obtained from single-cell genomes or metagenomes and values in parentheses are the 16S rRNA gene sequence similarity with those from single-cell genomes obtained in this study. Filled circles indicate that the corresponding nodes had >70% bootstrap values based on 1,000 resamplings. Ca. *Caldatribacterium californiense* that belongs to the OP9 lineage of Atribacteria was used as an outgroup.

Eighteen SAGs of the JS1 lineage from a sediment sample (40 cmbsf) were recovered by single-cell sorting and genome amplification. The size of each SAG ranged from 300 to 894 kb (Supplementary Table S3). The SAGs shared 16S rRNA gene sequence similarity ≥99.4% and had >99.5% 16S rRNA gene sequence similarity with B_OTU1, the most predominant JS1 OTU at this site (Supplementary Table S4). When 16S rRNA gene sequences from this study were compared with those retrieved from JS1 genomes from other habitats, SCGC_AD-561_N23 from the Adélie Basin offshore Antarctica showed the highest similarity (96.2–97.1%), followed by ASPA from Sakinaw Lake (93.2–93.9%) (Table 1 and Supplementary Table S4). Phylogenies inferred from 16S rRNA gene sequences revealed that the JS1 lineage obtained in this study formed a monophyletic clade (Figure 3). The ANI values between overlapping regions of the SAGs ranged from 96.8 to 99.9%, with an average of 99.2% per SAG (Supplementary Table S5), and this level was above the ANI cut-off value (95–96%) proposed for delineating bacterial species (Goris et al., 2007; Richter and Rosselló-Móra, 2009). In contrast, the ANI values between the SAGs in this study and JS1 genomes from other habitats ranged from 84.3 to 93.9% (Supplementary Table S5). Based on the high 16S rRNA gene sequence similarities, ANI values and a phylogenetic tree supporting that the 18 SAGs were from a single species, all data sets were co-assembled to construct a cSAG designated RS JS1-cSAG. After the jackknifing procedure to remove chimeric sequences generated during MDA (Marshall et al., 2012), the assembly size was ∼2.24 Mb, with 34.8% GC content (Tables 1, 2). The N50 contig size was 5,234 bases, and the largest contig size was 32,879 bases. The number of predicted protein-coding sequences was 2,189, and a total of 831 protein-coding genes were assigned to RAST subsystem categories (Table 2). A total of 113 CSCGs were identified using an HMM search against PFAM databases, and the estimated genome completeness of the RS JS1-cSAG was found to be 90.32% (Table 2 and Supplementary Table S6). Genome completeness and contamination estimated using CheckM were 85.5 and 1.7%, respectively (Table 2).

**Table 2:** Assembly statistics and genomic features of RS JS1-cSCG.

General features	RS JS1-cSCG
Assembly size (bp)	2,233,071
No. of contigs	698
Contig N50, bases	5,234
Largest contig bases	32,879
G+C content (%)	34.8
Protein coding genes	2,189
Function assigned	831
Hypothetical	645
rRNA genes	5S (1), 16S (2), and 23S (2)
tRNA genes	37
Genome completeness^1^	90.32%
Genome completeness/contamination^2^	85.5%/1.7%

*^1^Genome completeness was estimated using the Conserved Single Copy Gene set, as previously described by Rinke et al. (2013).*

*^2^Genome completeness and contamination were estimated using CheckM (Parks et al., 2015).*

### Genomic Features of RS JS1-cSAG

#### ABC Transporters

**FIGURE 4:**
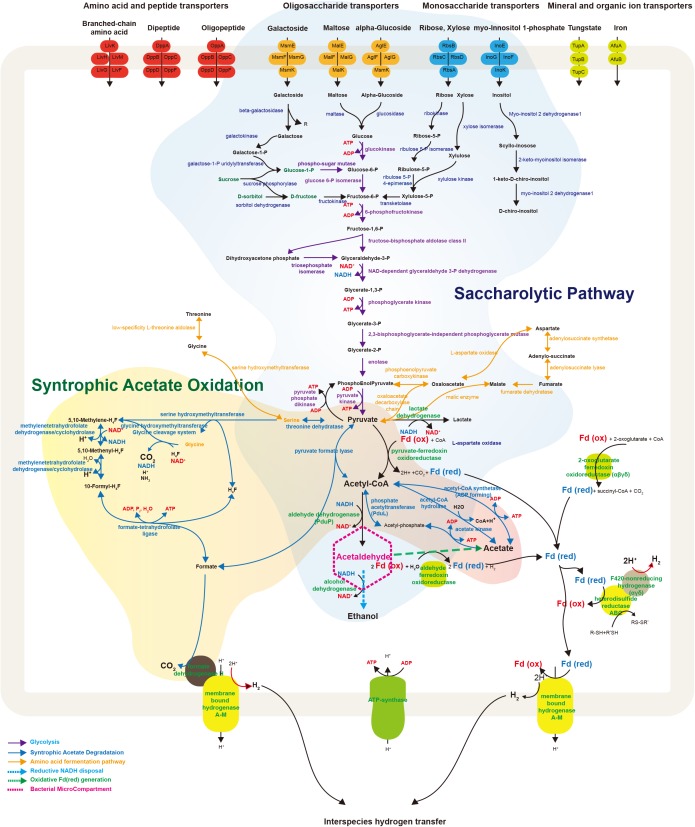
Schematic illustration representing metabolic and transport proteins hypothesized from the genome of *Ca.* Atribacteria JS1. ABC transporters for specific molecules in the bacterial cell membrane are marked by color: amino acid and peptide transporters (red); oligosaccharide transporters (orange); monosaccharide transporters (blue); and mineral and organic ion transporters (green). The degradative metabolism of oligosaccharides and monosaccharides and the syntrophic acetate oxidation pathway are annotated in detail. Proteins included in this schematic of genes encoded by the RS JS1-cSAG are listed in Supplementary File 2. CoA, coenzyme A; Fd, ferredoxin; Co, cobalamin; H_4_F, tetrahydrofolate; ATP, adenosine triphosphate; ADP, adenosine diphosphate.

ATP-binding cassette (ABC) transporters are a major class of cellular translocation machinery in all bacterial species and often consist of multiple subunits such as an ATP-binding protein, a membrane protein, and a substrate-binding protein. The RS JS1-cSAG encoded various ABC transporters for amino acids, peptides, oligosaccharides, monosaccharides, minerals, and organic ions (Figure 4). Multiple copies of genes in the RS JS1-cSAG assembly were annotated as ABC transporters of branched-chain amino acids, dipeptides, oligopeptides, ribose, and xylose. The RS JS1-cSAG had several unique transporters compared to other JS1 genomes, including those for maltose and galactoside (Supplementary Table S7).

#### Bacterial Microcompartments and Cell Envelope Structure

Bacterial microcompartments (BMCs) are organelles enveloped with a protein shell that can promote specific metabolic processes by encapsulating and colocalizing enzymes with their substrates and cofactors (Kerfeld et al., 2012; Nobu et al., 2016). Most BMCs share homologous shell proteins while the function of BMCs are different according to the genes adjacent to shell protein genes in BMC gene loci (Axen et al., 2014). The BMC gene loci of the RS JS1-cSAG were compared with those in the Sakinaw Lake SAG co-assembly and BMC genes present in the Sakinaw Lake SAG co-assembly were also found in the RS JS1-cSAG (Supplementary Table S8). In addition to genes encoding the shell proteins, coding sequences for deoxyribose-phosphate aldolase and ribose 5-phosphate isomerase were identified adjacent to shell protein genes in BMC gene loci implying that BMCs of the RS JS1-cSAG may be involved in aldehyde and sugar metabolism as suggested by Nobu et al. (2016). Another known feature of *Ca.* Atribacteria is the diderm cell envelope structure, which has an outer membrane (Dodsworth et al., 2013). HMM search results of protein-coding genes against PFAM databases revealed that some proteins involved in diderm cell structure, such as BamA for outer membrane protein assembly and TolC for type I secretin, were present in the RS JS1-cSAG (Supplementary Table S9).

#### Central Metabolism and Energy Conservation

The RS JS1-cSAG possesses genes involved in metabolism to convert various sugars transported into the cytosol via ABC transporters to pyruvate (Figure 4). Genes encoding ß-galactosidase, maltase, and glucosidase that cleave transported oligosaccharides such as galactoside, maltose, and α-glucoside into monosaccharides were found. The RS JS1-cSAG also encoded enzymes that convert other monosaccharides such as galactose, ribose and xylose into intermediates in glycolysis. A complete set of genes encoding the enzymes of glycolysis were identified. In addition, the RS JS1-cSAG also encoded enzymes that convert amino acids such as threonine, glycine, serine, and aspartate to pyruvate and *vice versa* (Supplementary Table S10). The RS JS1-cSAG possesses the potential to convert pyruvate to acetyl-CoA by pyruvate formate lyase or pyruvate-ferredoxin oxidoreductase reducing ferredoxin. Coding sequences for alcohol dehydrogenase and lactate dehydrogenase suggested lactate and ethanol production through the additional fermentation of pyruvate, using the reducing power generated in glycolysis (Figure 4).

The RS JS1-cSAG encodes the novel syntrophic acetate degradation pathway using tetrahydrofolate (THF) through the glycine cleavage system (Nobu et al., 2015; Figure 4). The RS JS1-cSAG has sequences for pyruvate formate lyase, which condenses acetyl-CoA produced from acetate by acetate kinase with formate to produce pyruvate, and threonine dehydrogenase, which aminates pyruvate to form serine. Genes encoding serine hydroxymethyltransferase, which splits serine into glycine and converts THF to 5,10-methylene-THF and the enzymes that oxidize 5,10-methylene-THF to yield formate were identified. Coding sequences for formate dehydrogenase H (FdhH) and membrane-bound hydrogenases (Mbh) A–M, which can oxidize formate to generate H_2_ and CO_2,_ were present in the RS JS1-cSAG. When Mbh extrudes protons outside the membrane for energy conservation, reduced ferredoxin supplies an electron and formate also can be used as an electron donor by FdhH (Figure 4). Electron-bifurcating hydrogenase-like genes encoding F420-non-reducing hydrogenase alpha, delta and gamma subunits, and heterodisulfide reductase (Hdr) genes encoding HdrABC, which may allow hydrogen production were identified in the RS JS1-cSAG (Figure 4).

## Discussion

### Dominance of Microbial Taxa Frequently Recovered From Methanogenic Environments

In this study, we observed a clear down-core shift in microbial community composition. In particular, several anaerobic lineages with no cultured representatives, such as Euryarchaeota and Lokiarchaeota in archaea and JS1 in bacteria, which have been found in almost methanogenic sediments, were abundant in subsurface sediments. Members of the class Thermoplasmata of Euryarchaeota are known to perform methylotrophic methanogenesis and degrade methylamines via a metatranscriptomic survey (Poulsen et al., 2013). The ecophysiological characteristics and metabolism of members of Lokiarchaeota are not well known owing to a lack of cultured representatives. Sequences belonging to this group have been frequently found in anaerobic and methanogenic sediments, such as gas hydrate-bearing sediments, mud volcanoes, and gas seep, implying that the members of Lokiarchaeota may be closely related to methane cycling (Teske and Sorensen, 2007; Parkes et al., 2014; Ruff et al., 2015). Metagenome sequence of Lokiarchaeota from the sediments near the Loki’s Castle hydrothermal vents of the Arctic Ocean revealed that it harbors a complete tetrahydromethanopterin (H_4_MPT)-dependent Wood-Ljungdahl pathway and enzymes revealing the hydrogen dependent potential (Sousa et al., 2016). In addition, in spite of the lack of almost all genes that are specific to methanogenesis, methanogen-like metabolism was suggested by the high similarity of enzymes of H_4_MPT-dependent Wood-Ljungdahl pathway to methanogen homologs (Sousa et al., 2016). Members of the JS1 lineage have been recovered frequently from strictly anoxic organic-rich and/or methanogenic environments and specialize in either primary fermentation of carbohydrates or secondary fermentation of organic acids, such as acetate or propionate (Inagaki et al., 2006; Webster et al., 2006a; Blazejak and Schippers, 2010; Orcutt et al., 2011; Gies et al., 2014; Kaster et al., 2014; Parkes et al., 2014; Carr et al., 2015; Nobu et al., 2015, 2016; Oni et al., 2015; Fullerton and Moyer, 2016; Saxton et al., 2016). Methane concentrations were not analyzed in this study and only a few known methanogens (less than 1%) were observed. Thus, the presence of methane in the study area should be inferred with caution. However, environments where a significant fraction of methane is produced appear to only harbor small fraction of methanogen populations (Inagaki et al., 2006; Gies et al., 2014; Lever, 2016; Carr et al., 2018). Additionally, seismic evidence of gas hydrates and free gas have been reported in the Victoria Land Basin and pockmarks off Franklin Island in the Ross Sea, indicating the potential of subsurface gas release in the study area (Geletti and Busetti, 2011; Lawver et al., 2012). Therefore, the presence of microbial groups frequently found in gas hydrates, organic-rich sediments, and other methanogenic environments and seismic surveys seem to suggest the possibility of biogenic methane production in the sediments of the western Ross Sea.

### Metabolic Potential Supporting the Predominance of the JS1 Lineage in Methanogenic Marine Environments

The genomes of JS1 obtained in this study showed >99.5% 16S rRNA gene similarity with the predominant JS1 OTU throughout the core and formed a monophyletic clade, indicating that these genomes represent the dominant JS1 species of the study area. Because the 18 SAGs obtained in this study were considered to be a single species, a genome, RS JS1-cSAG with the most complete JS1 metabolic information within an environment could be obtained by co-assembly of the 18 SAGs. RS JS1-cSAG was classified into “medium-quality” draft genome according to the criteria suggested by Bowers et al. (2017). The genomes in our study showed their highest 16S rRNA gene similarity with that of SCGC AD-561-N23 from marine sediment of the Adélie Basin (Carr et al., 2015). While SCGC AD-561-N23 was the first reported JS1 genome from Antarctic marine sediment, its low level of completeness limited the metabolic interpretation and the detailed prediction of *in situ* ecological functions of the lineage (Carr et al., 2015). However, the high genome completeness of the predominant JS1 species throughout the sediments in this study provides additional insight into the metabolic pathways and fundamental lifestyle of this bacterium in anaerobic sediments. A predominant JS1 species from the Ross Sea (RS JS1) showed metabolic potential for fermentation of pyruvate produced from various sugars and amino acids in anaerobic environments. Further supporting evidence of fermentation, the RS JS1-cSAG was found to harbor ABC transporters for various oligosaccharides, monosaccharides, and amino acids, and enzymes involved in glycolysis and fermentation. In particular, RS JS1-cSAG contained unique ABC transporters for maltose, and additional copies of ABC transporters for branched-chain amino acids, dipeptides and oligopeptides compared to other JS1 genomes. The presence of coding sequences for various ABC transporters together with complete gene sets for glycolysis and fermentation seemed to indicate the high potential of RS JS1 to use various substrates to gain energy via fermentation producing H_2_, CO_2_, acetate, and formate as suggested for JS1 genomes from Sakinaw lake, marine sediment of the Adélie Basin, and methanogenic bioreactor (Gies et al., 2014; Carr et al., 2015; Nobu et al., 2015). These products can serve as substrates for methanogenesis and partially explains the importance of fermenters to methanogenic communities (Sieber et al., 2012).

The RS JS1-cSAG also showed the possibility for syntrophic association with hydrogenotrophic methanogens via acetate oxidation. Acetate oxidation, which is thermodynamically unfavorable and thus cannot proceed without hydrogen or formate-scavenging methanogens, can occur through syntrophic association (Hattori, 2008; Stams and Plugge, 2009). The syntrophic acetate-oxidizing bacterium, *Pseudothermotoga lettingae* strain TMO is suggested to degrade acetate using the THF pathway, whereas *Clostridium ultunense* strains BS and AOR grow in syntrophic mode with hydrogenotrophic methanogens by running the Wood-Ljungdahl pathway in reverse (Lee and Zinder, 1988; Schnurer et al., 1996; Hattori, 2008). However, the RS JS1-cSAG did not encode carbon monoxide dehydrogenase/acetyl-CoA synthase, the key enzyme that produces an acetyl moiety from the methyl and carbonyl groups, finally producing acetyl-CoA in the Wood-Ljungdahl pathway (Supplementary Figure S4). Consistent with this, carbon monoxide dehydrogenase/acetyl-CoA synthase complex has not been identified in other JS1 genomes (Dodsworth et al., 2013; Lloyd et al., 2013; Rinke et al., 2013; Carr et al., 2015; Nobu et al., 2016). Thus, the syntrophic acetate oxidation by running the Wood-Ljungdahl pathway in reverse seems to be unlikely in JS1 bacteria based on their genomic information. Instead, RS JS1 has potential to perform acetate oxidation via a novel syntrophic acetate degradation pathway through the glycine cleavage system in a syntrophic association with hydrogenotrophic methanogens (Nobu et al., 2015). Although correlation results should be interpreted with caution due to the small number of samples compared, Spearman correlation coefficients between the most dominant JS1 OTU and major archaeal OTUs (≥3% in relative abundance) were calculated to investigate whether methanogens in this environment are related with JS1 species. One archaeal OTU belongs to Lokiarchaeota, which was mainly found in methanogenic areas such as the Ulleung Basin of the East Sea, the Okhotsk Sea, the Shimokita Peninsula of Japan, and the Gulf of Mexico, showed a covariation pattern with the most dominant JS1 OTU. However, the low 16S rRNA gene similarity of the archaeal OTU with sequences of known strains and the lack of most genes that are specific to methanogenesis in the previously known Lokiarchaeota genome (Sousa et al., 2016) made it difficult to infer their potential as hydrogenotrophic methanogens and for syntrophic association with JS1. Hydrogen produced via fermentation of various sugars and amino acids seems to support the correlation between JS1 and hydrogen dependent Lokiarchaeota. In addition, while no correlations with known methanogens of Euryarchaeota were identified in this dataset, considering the presence of phylogenetically widespread methanogens with low similarity of methanogenesis-involved gene sequences as suggested by Lever (2016), it does not rule out that syntrophic relationship between JS1 lineage and unknown methanogens might exist explaining the predominance of the JS1 lineage in methanogenic environments.

**FIGURE 5:**
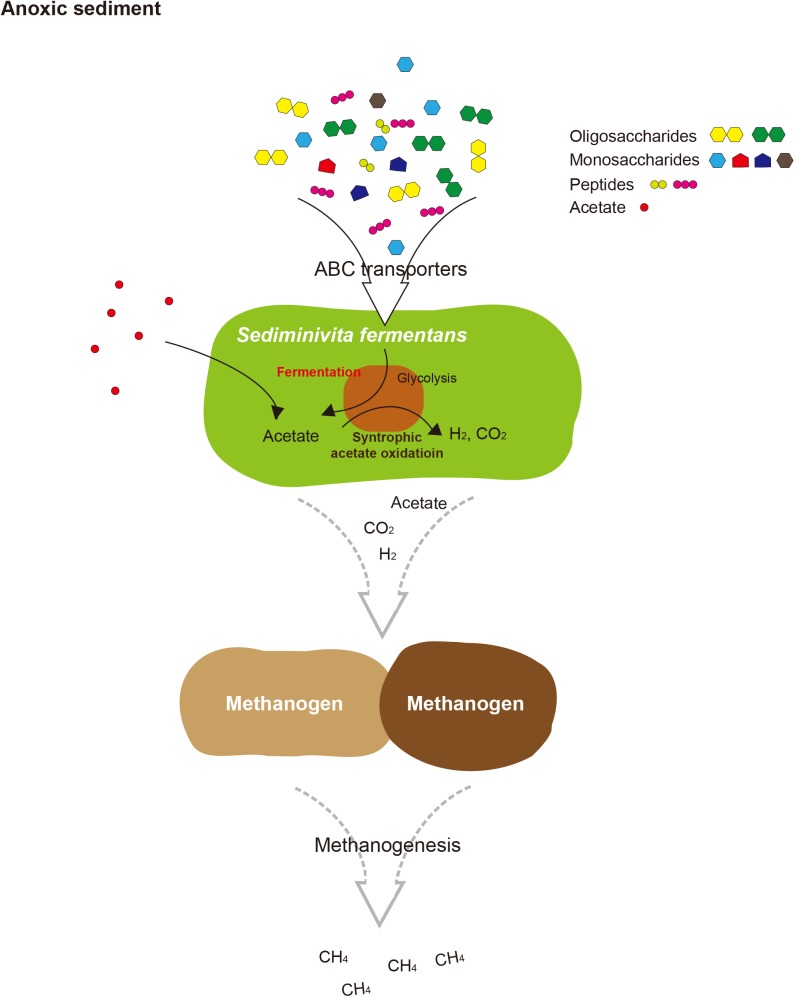
Proposed schematic interactions of *Sediminivita fermentans* with methanogens and importance in carbon cycling. *Sediminivita fermentans* ferments pyruvate produced in glycolysis of various amino acids and sugars producing acetate, and also oxidizes acetate producing hydrogen and carbon dioxide via syntrophic association with hydrogen-scavenging methanogens. The resulting acetate, hydrogen and carbon dioxide can support methanogenesis in anoxic sediments.

The BMCs function both in syntrophic metabolism and sugar or amino acid fermentation by Atribacteria was known from genome analysis (Nobu et al., 2016). In *Ca*. Caldatribacterium of OP9 lineages and genomes of JS1 lineages from a meromictic lake and bioreactor, NADH produced from sugar oxidation or propionate degradation is consumed by aldehyde dehydrogenase which reduces acetyl-CoA to acetaldehyde within the BMC (Nobu et al., 2016). The RS JS1-cSAG encoded sequences of aldehyde dehydrogenase, supporting the potential for a BMC-associated NADH sink (Supplementary Table S8 and Figure 4). The BMC gene loci of RS JS1 also encoded deoxyribose-phosphate aldolase and ribose 5-phosphate isomerase facilitating aldehyde condensation and storage. Unlike other OP9 and JS1 lineages, the stored carbon within the BMC of the RS JS1-cSAG could be used both for reductive NADH sink via alcohol dehydrogenase, and for oxidative Fd_red_ generation, which could allow H_2_ generation by Mbh regardless of exogenous substrates through aldehyde:Fd oxidoreductases in the cytosol (Nobu et al., 2016). Thus, BMCs in the RS JS1-cSAG could control sugar fermentation and interact with syntrophic association by facilitating H_2_ generation via Mbh. This flexibility of carbon storage and later use as an electron sink or source in RS JS1 seems to be advantageous in terms of provision of methanogenic substrates such as hydrogen, CO_2,_ and formate via fermentation of various sugars and amino acids and syntrophic acetate degradation, explaining the predominance of the JS1 lineage in methanogenic environments. Taken together, a hypothetical metabolic process of RS JS1 in anaerobic marine sediment of the Ross Sea is illustrated in Figure 5.

### Proposal of *Candidatus* Sediminivita Fermentans to Refer the Predominant JS1 Species in Marine Sediment of the Ross Sea

In this study, the most complete JS1 genome to date was obtained from the predominant species in marine sediment of the Ross Sea. Based on the data presented here, we propose the name “*Candidatus* Sediminivita fermentans” to refer RS JS1. The description of the taxon name is: “*Sediminivita*” (Se.di.mi.ni.vi’ta. N.L. n. *sedimen* sediment; L. fem. n. *vita* life; N.L. fem. n. *Sediminivita* fermentans) (fer.men’tans. L. part. adj. *fermentans* fermenting). The genome of *Candidatus* Sediminivita fermentans revealed metabolic potential as a fermenter using various substrates and a syntrophic acetate oxidizer. Although it is still not clear which lifestyle is predominant in various environments, *Candidatus* Sediminivita fermentans seems to have the potential for increased survival in nature by using various heterotrophic substrates to outcompete other primary and secondary fermenters, or through syntrophic interactions with partner methanogens. This growth mode may explain the dominance of *Candidatus* Sediminivita fermentans within anoxic sediments, implying an important ecological function of *Candidatus* Sediminivita fermentans in carbon cycling.

## Author Contributions

YL, SH, SS, HL, and JC conceived and designed the experiments. YL, conducted the experiments for microbial diversity. SH performed the 16S rRNA phylogenetic analysis. YL, KH, MK, and CH analyzed the pyrosequencing sequences. KH analyzed the isolation origin. JL and H-JN collected and processed the samples. JL and HC analyzed the environmental data. SS analyzed the single-cell genomes. YL and SS wrote the paper with input and approval by all authors.

## Conflict of Interest Statement

The authors declare that the research was conducted in the absence of any commercial or financial relationships that could be construed as a potential conflict of interest.

## Footnotes

1 http://pfam.xfam.org/

2 http://rast.nmpdr.org/

## References

[B1] Axen S. D. Erbilgin O. Kerfeld C. A. (2014). A taxonomy of bacterial microcompartment loci constructed by a novel scoring method. *PLoS Comput. Biol.* 10:e1003898. 10.1371/journal.pcbi.1003898 25340524 PMC4207490

[B2] Aziz R. K. Bartels D. Best A. A. Dejongh M. Disz T. Edwards R. A. (2008). The RAST Server: rapid annotations using subsystems technology. *BMC Genomics* 9:75. 10.1186/1471-2164-9-75 18261238 PMC2265698

[B3] Blazejak A. Schippers A. (2010). High abundance of JS-1- and Chloroflexi-related bacteria in deeply buried marine sediments revealed by quantitative, real-time PCR. *FEMS Microbiol. Ecol.* 72 198–207. 10.1111/j.1574-6941.2010.00838.x 20180854

[B4] Bowers R. M. Kyrpides N. C. Stepanauskas R. Harmon-Smith M. Doud D. Reddy T. B. K. (2017). Minimum information about a single amplified genome (MISAG) and a metagenome-assembled genome (MIMAG) of bacteria and archaea. *Nat. Biotechnol.* 35 725–731. 10.1038/nbt.3893 28787424 PMC6436528

[B5] Carr S. A. Orcutt B. N. Mandernack K. W. Spear J. R. (2015). Abundant Atribacteria in deep marine sediment from the Adélie Basin, Antarctica. *Front. Microbiol.* 6:872 10.3389/fmicb.2015.00872 PMC4549626 26379647

[B6] Carr S. A. Schubotz F. Dunbar R. B. Mills C. T. Dias R. Summons R. E. (2018). Acetoclastic Methanosaeta are dominant methanogens in organic-rich Antarctic marine sediments. *ISME J.* 12 330–342. 10.1038/ismej.2017.150 29039843 PMC5776447

[B7] Clarke K. Gorley R. (2006). *PRIMER v6: User Manual/Tutorial (Plymouth Routines in Multivariate Ecological Research).* Plymouth: Primer-E Ltd.

[B8] Corinaldesi C. (2015). New perspectives in benthic deep-sea microbial ecology. *Front. Mar. Sci.* 2:17 10.3389/fmars.2015.00017

[B9] Dodsworth J. A. Blainey P. C. Murugapiran S. K. Swingley W. D. Ross C. A. Tringe S. G. (2013). Single-cell and metagenomic analyses indicate a fermentative and saccharolytic lifestyle for members of the OP9 lineage. *Nat. Commun.* 4:1854. 10.1038/ncomms2884 23673639 PMC3878185

[B10] Edgar R. C. Haas B. J. Clemente J. C. Quince C. Knight R. (2011). UCHIME improves sensitivity and speed of chimera detection. *Bioinformatics* 27 2194–2200. 10.1093/bioinformatics/btr381 21700674 PMC3150044

[B11] Eloe-Fadrosh E. A. Ivanova N. N. Woyke T. Kyrpides N. C. (2016). Metagenomics uncovers gaps in amplicon-based detection of microbial diversity. *Nat. Microbiol.* 1:15032. 10.1038/nmicrobiol.2015.32 27572438

[B12] Felsenstein J. (1981). Evolutionary trees from DNA sequences: a maximum likelihood approach. *J. Mol. Evol.* 17 368–376. 10.1007/BF01734359 7288891

[B13] Finn R. D. Coggill P. Eberhardt R. Y. Eddy S. R. Mistry J. Mitchell A. L. (2016). The Pfam protein families database: towards a more sustainable future. *Nucleic Acids Res.* 44 D279–D285. 10.1093/nar/gkv1344 26673716 PMC4702930

[B14] Fullerton H. Moyer C. L. (2016). Comparative single-cell genomics of *Chloroflexi* from the Okinawa trough deep-subsurface biosphere. *Appl. Environ. Microbiol.* 82 3000–3008. 10.1128/AEM.00624-16 26969693 PMC4959059

[B15] Geletti R. Busetti M. (2011). A double bottom simulating reflector in the western Ross Sea, Antarctica. *J. Geophys. Res* 116: B04101. 10.1128/AEM.00624-16 26969693 PMC4959059

[B16] Gies E. A. Konwar K. M. Beatty J. T. Hallam S. J. (2014). Illuminating microbial dark matter in meromictic Sakinaw Lake. *Appl. Environ. Microbiol.* 80 6807–6818. 10.1128/AEM.01774-14 25172853 PMC4249029

[B17] Goris J. Konstantinidis K. T. Klappenbach J. A. Coenye T. Vandamme P. Tiedje J. M. (2007). DNA–DNA hybridization values and their relationship to whole-genome sequence similarities. *Int. J. Syst. Evol. Microbiol.* 57 81–91. 10.1099/ijs.0.64483-0 17220447

[B18] Hamdan L. J. Coffin R. B. Sikaroodi M. Greinert J. Treude T. Gillevet P. M. (2012). Ocean currents shape the microbiome of Arctic marine sediments. *ISME J.* 7 685–696. 10.1038/ismej.2012.143 23190727 PMC3603395

[B19] Handelsman J. (2004). Metagenomics: application of genomics to uncultured microorganisms. *Microbiol. Mol. Biol. Rev.* 68 669–685. 10.1128/MMBR.68.4.669-685.2004 15590779 PMC539003

[B20] Hattori S. (2008). Syntrophic acetate-oxidizing microbes in methanogenic environments. *Microbes Environ.* 23 118–127. 10.1264/jsme2.23.118 21558697

[B21] Hugenholtz P. Pitulle C. Hershberger K. L. Pace N. R. (1998). Novel division level bacterial diversity in a Yellowstone hot spring. *J. Bacteriol.* 180 366–376. 9440526 10.1128/jb.180.2.366-376.1998 PMC106892

[B22] Hwang K. Oh J. Kim T.-K. Kim B. K. Yu D. S. Hou B. K. (2013). CLUSTOM: a novel method for clustering 16S rRNA next generation sequences by overlap minimization. *PLoS One* 8:e62623. 10.1371/journal.pone.0062623 23650520 PMC3641076

[B23] Inagaki F. Nunoura T. Nakagawa S. Teske A. Lever M. Lauer A. (2006). Biogeographical distribution and diversity of microbes in methane hydrate-bearing deep marine sediments on the Pacific Ocean Margin. *Proc. Natl. Acad. Sci. U.S.A.* 103 2815–2820. 10.1073/pnas.0511033103 16477011 PMC1413818

[B24] Jeon Y. S. Chung H. Park S. Hur I. Lee J. H. Chun J. (2005). jPHYDIT: a JAVA-based integrated environment for molecular phylogeny of ribosomal RNA sequences. *Bioinformatics* 21 3171–3173. 10.1093/bioinformatics/bti463 15855247

[B25] Jorgensen S. L. Hannisdal B. Lanzén A. Baumberger T. Flesland K. Fonseca R. (2012). Correlating microbial community profiles with geochemical data in highly stratified sediments from the Arctic Mid-Ocean Ridge. *Proc. Natl. Acad. Sci. U.S.A.* 109 E2846–E2855. 10.1073/pnas.1207574109 23027979 PMC3479504

[B26] Kallmeyer J. Pockalny R. Adhikari R. R. Smith D. C. D’hondt S. (2012). Global distribution of microbial abundance and biomass in subseafloor sediment. *Proc. Natl. Acad. Sci. U.S.A.* 109 16213–16216. 10.1073/pnas.1203849109 22927371 PMC3479597

[B27] Kaster A.-K. Mayer-Blackwell K. Pasarelli B. Spormann A. M. (2014). Single cell genomic study of Dehalococcoidetes species from deep-sea sediments of the Peruvian Margin. *ISME J.* 8 1831–1842. 10.1038/ismej.2014.24 24599070 PMC4139717

[B28] Kato C. Li L. Tamaoka J. Horikoshi K. (1997). Molecular analyses of the sediment of the 11000-m deep Mariana Trench. *Extremophiles* 1 117–123. 10.1007/s007920050024 9680317

[B29] Kerfeld C. A. Heinhorst S. Cannon G. C. (2012). Bacterial microcompartments. *Ann. Rev. Microbiol.* 64 391–408. 10.1146/annurev.micro.112408.134211 20825353

[B30] Kim O.-S. Cho Y.-J. Lee K. Yoon S.-H. Kim M. Na H. (2012). Introducing EzTaxon-e: a prokaryotic 16S rRNA gene sequence database with phylotypes that represent uncultured species. *Int. J. Syst. Evol. Microbiol.* 62 716–721. 10.1099/ijs.0.038075-0 22140171

[B31] Kurtz S. Phillippy A. Delcher A. L. Smoot M. Shumway M. Antonescu C. (2004). Versatile and open software for comparing large genomes. *Genome Biol.* 5:R12. 10.1186/gb-2004-5-2-r12 14759262 PMC395750

[B32] Lane D. J. (1991). “16S/23S rRNA sequencing,” in *Nucleic Acid Techniques in Bacterial Systematics*, eds Stackebrandt E. Goodfellow M. (New York, NY: John Wiley and Sons), 115–175.

[B33] Lawver L. Lee J. Kim Y. Davey F. (2012). Flat-topped mounds in western Ross Sea: carbonate mounds or subglacial volcanic features? *Geosphere* 8 645–653. 10.1130/GES00766.1

[B34] Lee M. J. Zinder S. H. (1988). Carbon monoxide pathway enzyme activities in a thermophilic anaerobic bacterium grown acetogenically and in a syntrophic acetate-oxidizing coculture. *Arch. Microbiol.* 150 513–518. 10.1007/BF00408241

[B35] Lee Y. M. (2017). *Study in the Diversity and Ecological Functions of Bacterial Communities in the Southern Ocean.* Ph.D. thesis, Seoul National University, Seoul.

[B36] Lever M. A. (2016). A new era of methanogenesis research. *Trends Micrbiol.* 24 84–86. 10.1016/j.tim.2015.12.005 26765541

[B37] Lloyd K. G. Schreiber L. Petersen D. G. Kjeldsen K. U. Lever M. A. Steen A. D. (2013). Predominant archaea in marine sediments degrade detrital proteins. *Nature* 496 215–218. 10.1038/nature12033 23535597

[B38] Macaulay I. C. Voet T. (2014). Single cell genomics: advances and future perspectives. *PLoS Genet.* 10:e1004126. 10.1371/journal.pgen.1004126 24497842 PMC3907301

[B39] Marshall I. P. Blainey P. C. Spormann A. M. Quake S. R. (2012). A single-cell genome for Thiovulum sp. *Appl. Environ. Microbiol.* 78 8555–8563. 10.1128/AEM.02314-12 23023751 PMC3502928

[B40] Moriya Y. Itoh M. Okuda S. Yoshizawa A. C. Kanehisa M. (2007). KAAS: an automatic genome annotation and pathway reconstruction server. *Nucleic Acids Res.* 35 W182–W185. 10.1093/nar/gkm321 17526522 PMC1933193

[B41] Nei M. Kumar S. (2000). *Molecular Evolution and Phylogenetics.* New York, NY: Oxford University Press.

[B42] Nobu M. K. Dodsworth J. A. Murugapiran S. K. Rinke C. Gies E. A. Webster G. (2016). Phylogeny and physiology of candidate phylum ’Atribacteria’ (OP9/JS1) inferred from cultivation-independent genomics. *ISME J.* 10 273–286. 10.1038/ismej.2015.97 26090992 PMC4737943

[B43] Nobu M. K. Narihiro T. Rinke C. Kamagata Y. Tringe S. G. Woyke T. (2015). Microbial dark matter ecogenomics reveals complex synergistic networks in a methanogenic bioreactor. *ISME J.* 9 1710–1722. 10.1038/ismej.2014.256 25615435 PMC4511927

[B44] Nunoura T. Takaki Y. Shimamura S. Kakuta J. Kazama H. Hirai M. (2016). Variance and potential niche separation of microbial communities in subseafloor sediments off Shimokita Peninsula. *Jpn. Environ. Microbiol.* 18 1889–1906. 10.1111/1462-2920.13096 26486095

[B45] Oh J. Kim B. K. Cho W.-S. Hong S. G. Kim K. M. (2012). PyroTrimmer: a Software with GUI for pre-processing 454 amplicon sequences. *J. Microbiol.* 50 766–769. 10.1007/s12275-012-2494-6 23124743

[B46] Oni O. E. Schmidt F. Miyatake T. Kasten S. Witt M. Hinrichs K.-U. (2015). Microbial communities and organic matter composition in surface and subsurface sediments of the Helgoland Mud Area, North Sea. *Front. Microbiol.* 6:1290. 10.3389/fmicb.2015.01290 26635758 PMC4658423

[B47] Orcutt B. N. Sylvan J. B. Knab N. J. Edwards K. J. (2011). Microbial ecology of the dark ocean above, at, and below the seafloor. *Microbiol. Mol. Biol. Rev.* 75 361–422. 10.1128/MMBR.00039-10 21646433 PMC3122624

[B48] Parkes R. J. Cragg B. Roussel E. Webster G. Weightman A. Sass H. (2014). A review of prokaryotic populations and processes in sub-seafloor sediments, including biosphere:geosphere interactions. *Mar. Geol.* 352 409–425. 10.1016/j.margeo.2014.02.009

[B49] Parks D. H. Imelfort M. Skennerton C. T. Hugenholtz P. Tyson G. W. (2015). CheckM:assessing the quality of microbial genomes recovered from isolates, single cells, and metagenomes. *Genome Res.* 25 1043–1055. 10.1101/gr.186072.114 25977477 PMC4484387

[B50] Poulsen M. Schwab C. Borg Jensen B. Engberg R. M. Spang A. Canibe N. (2013). Methylotrophic methanogenic Thermoplasmata implicated in reduced methane emissions from bovine rumen. *Nat. Commun.* 4:1428. 10.1038/ncomms2432 23385573

[B51] Richter M. Rosselló-Móra R. (2009). Shifting the genomic gold standard for the prokaryotic species definition. *Proc. Natl. Acad. Sci. U.S.A.* 106 19126–19131. 10.1073/pnas.0906412106 19855009 PMC2776425

[B52] Rinke C. Schwientek P. Sczyrba A. Ivanova N. N. Anderson I. J. Cheng J.-F. (2013). Insights into the phylogeny and coding potential of microbial dark matter. *Nature* 499 431–437. 10.1038/nature12352 23851394

[B53] Rochelle P. A. Cragg B. A. Fry J. C. Parkes R. J. Weightman A. J. (1994). Effect of sample handling on estimation of bacterial diversity in marine sediments by 16S rRNA gene sequence analysis. *FEMS Microbiol. Ecol.* 15 215–225. 10.1111/j.1574-6941.1994.tb00245.x

[B54] Ruff S. E. Biddle J. F. Teske A. P. Knittel K. Boetius A. Ramette A. (2015). Global dispersion and local diversification of the methane seep microbiome. *Proc. Natl. Acad. Sci. U.S.A.* 112 4015–4020. 10.1073/pnas.1421865112 25775520 PMC4386351

[B55] Saxton M. A. Samarkin V. A. Schutte C. A. Bowles M. W. Madigan M. T. Cadieux S. B. (2016). Biogeochemical and 16S rRNA gene sequence evidence supports a novel mode of anaerobic methanotrophy in permanently ice-covered Lake Fryxell, Antarctica. *Limnol. Oceanogr.* 61 S119–S130. 10.1002/lno.10320

[B56] Schauer R. Bienhold C. Ramette A. Harder J. (2009). Bacterial diversity and biogeography in deep-sea surface sediments of the South Atlantic Ocean. *ISME J.* 4 159–170. 10.1038/ismej.2009.106 19829317

[B57] Schnurer A. Schink B. Svensson B. H. (1996). Clostridium ultunense sp. nov., a mesophilic bacterium oxidizing acetate in syntrophic association with a hydrogenotrophic methanogenic bacterium. *Int. J. Syst. Evol. Microbiol.* 46 1145–1152. 10.1099/00207713-46-4-1145 8863449

[B58] Sieber J. R. Mcinerney M. J. Gunsalus R. P. (2012). Genomic insights into syntrophy: the paradigm for anaerobic metabolic cooperation. *Ann. Rev. Microbiol.* 66 429–452. 10.1146/annurev-micro-090110-102844 22803797

[B59] Sousa F. L. Neukirchen S. Allen J. F. Lane N. Martin W. F. (2016). Lokiarchaeon is hydrogen dependent. *Nat. Microbiol.* 1 1–3. 10.1038/nmicrobiol.2016.34 27572645

[B60] Stams A. J. M. Plugge C. M. (2009). Electron transfer in syntrophic communities of anaerobic bacteria and archaea. *Nat. Rev. Microbiol.* 7 568–577. 10.1038/nrmicro2166 19609258

[B61] Tamura K. Stecher G. Peterson D. Filipski A. Kumar S. (2013). MEGA6: molecular evolutionary genetics analysis version 6.0. *Mol. Biol. Evol.* 30 2725–2729. 10.1093/molbev/mst197 24132122 PMC3840312

[B62] Teske A. Sorensen K. B. (2007). Uncultured archaea in deep marine subsurface sediments: have we caught them all? *ISME J.* 2 3–18. 10.1038/ismej.2007.90 18180743

[B63] Webster G. Parkes R. J. Cragg B. A. Newberry C. J. Weightman A. J. Fry J. C. (2006a). Prokaryotic community composition and biogeochemical processes in deep subseafloor sediments from the Peru Margin. *FEMS Microbiol. Ecol.* 58 65–85. 10.1111/j.1574-6941.2006.00147.x 16958909

[B64] Webster G. Parkes R. J. Fry J. C. Weightman A. J. (2004). Widespread occurrence of a novel division of bacteria identified by 16S rRNA gene sequences originally found in deep marine sediments. *Appl. Environ. Microbiol.* 70 5708–5713. 10.1128/AEM.70.9.5708-5713.2004 15345467 PMC520855

[B65] Webster G. Sass H. Cragg B. A. Gorra R. Knab N. J. Green C. J. (2011). Enrichment and cultivation of prokaryotes associated with the sulphate–methane transition zone of diffusion-controlled sediments of Aarhus Bay, Denmark, under heterotrophic conditions. *FEMS Microbiol. Ecol.* 77 248–263. 10.1111/j.1574-6941.2011.01109.x 21477007

[B66] Webster G. Watt L. C. Rinna J. Fry J. C. Evershed R. P. Parkes R. J. (2006b). A comparison of stable-isotope probing of DNA and phospholipid fatty acids to study prokaryotic functional diversity in sulfate-reducing marine sediment enrichment slurries. *Environ. Microbiol.* 8 1575–1589. 16913918 10.1111/j.1462-2920.2006.01048.x

[B67] Zinger L. Amaral-Zettler L. A. Fuhrman J. A. Horner-Devine M. C. Huse S. M. Welch D. B. M. (2011). Global patterns of bacterial beta-diversity in seafloor and seawater ecosystems. *PLoS One* 6:e24570. 10.1371/journal.pone.0024570 21931760 PMC3169623

