# Supplementary material for: Genomic Insight Into the Predominance of Candidate Phylum Atribacteria JS1 Lineage in Marine Sediments
Source: Front Microbiol. 2018 Nov 29;9:2909. doi: 10.3389/fmicb.2018.02909 (PMC6281690; doi:10.3389/fmicb.2018.02909)
Supplement: Supplementary file 1 [file Table_1.DOCX]

**Table S1. Spearman correlation coefficients between the dominant OTU of JS1 and major archaeal OTUs**

| **OTU ID** | **B_OTU1** | |
| --- | --- | --- |
|  | **r*** | ***p*** |
| A_OTU6 | 0.31 | 0.544 |
| A_ OTU12 | 0.09 | 0.872 |
| A_ OTU10 | -0.26 | 0.623 |
| A_OTU29 | 0.26 | 0.623 |
| A_OTU5 | 0.71 | 0.111 |
| A_OTU8 | 0.37 | 0.468 |
| A_OTU11 | 0.26 | 0.623 |
| A_OTU18 | 0.09 | 0.872 |
| A_OTUL2 | 0.31 | 0.544 |
| **A_OTU15** | **0.94** | **0.005** |
| A_OTU9 | -0.03 | 0.957 |
| A_OTU4 | -0.60 | 0.208 |
| A_OTU1 | -0.49 | 0.329 |
| A_OTU3 | -0.83 | 0.042 |
| A_OTU7 | -0.65 | 0.158 |

* r indicates the Spearman correlation coefficient and *p* is the *p* value.

Bold indicates the archaeal OTU with significant positive correlation (p < 0.1) with a JS1 OTU, B_OTU1.

Major archaeal OTUs are OTUs with and more than 3% portion of archaeal communities.

**Table S2. The habitats of similar sequences with archaeal OTU**

| **OTU ID** | **Origins of environmental sequences^†^** | | | | | | | |
| --- | --- | --- | --- | --- | --- | --- | --- | --- |
|  | **Total** † | **Marine sediments**  **(Methanogenic *)** | **Sea water** | **Mangrove sediments** | **Lake sediment** | **Water from lake** | **Manganese nodule** | **Others**^§^ |
| A_OTU15^※^ | 131 | 106 (90) | 0 | 2 | 15 | 2 | 0 | 6 |

^※^ indicates archaeal OTU with positive and significant correlation with JS1 OTU, B_OTU1.

^‡^ Sequences with 97% or higher sequence similarity with A_OTU15 were retrieved by BLAST search in the GenBank database, and their habitat information was parsed from the database information.

† Total indicates the number of sequences with 97% or higher sequence similarity with each major OTU by BLAST search in the GenBank database.

* Methanogenic indicates methanogenic marine sediments such as gas hydrate-bearing sediments, mud volcanos, and gas seep from Ulleung Basin of East Sea, Okhotsk Sea, Shimokita Peninsula of Japan, and Gulf of Mexico.

^§^ Others include basalt, biofilm, cave, chimney, soil, ground water, and organisms.

**Table S3. Genome characteristics of single amplified genomes (SAGs) from Atribacteria**

|  | **SAG ID** | **Total reads** | **# reads assembled** | **Total # contigs** | **N50 contig size (bp)** | **Max contig size (bp)** | **Assembly size (bp)** | **G+C contents (%)** |
| --- | --- | --- | --- | --- | --- | --- | --- | --- |
| 1 | AG183N14 | 3,525,408 | 3,112,864 | 72 | 6,598 | 18,407 | 300,007 | 34.9 |
| 2 | AG183J17 | 2,786,182 | 2,727,664 | 407 | 1,461 | 25,397 | 335,029 | 36 |
| 3 | AG183D10 | 3,785,158 | 3,656,556 | 452 | 1,348 | 30,580 | 361,811 | 36.6 |
| 4 | AG183P08 | 4,114,504 | 3,642,932 | 74 | 8,880 | 26,459 | 369,086 | 37.7 |
| 5 | AG183P14 | 4,527,826 | 4,067,252 | 99 | 6,589 | 23,709 | 435,436 | 33.7 |
| 6 | AG183P13 | 2,987,058 | 2,917,108 | 450 | 3,980 | 32,609 | 456,911 | 35.6 |
| 7 | AG183D19 | 5,005,792 | 4,467,434 | 108 | 8,012 | 32,374 | 458,549 | 34.3 |
| 8 | AG183B19 | 4,432,318 | 3,961,380 | 122 | 6,200 | 21,868 | 504,934 | 36.9 |
| 9 | AG183A05 | 3,981,694 | 3,533,168 | 112 | 10,286 | 35,313 | 604,612 | 33.4 |
| 10 | AG183M21 | 3,741,560 | 3,629,894 | 633 | 3,269 | 26,741 | 653,913 | 35.1 |
| 11 | AG183O15 | 3,515,540 | 3,419,538 | 561 | 3,857 | 35,945 | 657,801 | 35.5 |
| 12 | AG183G20 | 3,345,186 | 2,916,062 | 156 | 7,062 | 24,763 | 664,301 | 33.8 |
| 13 | AG183C13 | 3,478,788 | 3,388,806 | 548 | 5,036 | 25,965 | 667,274 | 35.3 |
| 14 | AG183F16 | 3,139,342 | 3,050,738 | 611 | 3,072 | 26,157 | 675,445 | 35 |
| 15 | AG183P18 | 4,239,382 | 3,607,000 | 136 | 13,521 | 64,226 | 751,925 | 31.3 |
| 16 | AG183A11 | 2,843,454 | 2,757,232 | 723 | 3,433 | 19,535 | 763,790 | 34.9 |
| 17 | AG183N04 | 3,343,960 | 3,112,864 | 756 | 3,323 | 32,857 | 826,550 | 35.5 |
| 18 | AG183P03 | 3,010,548 | 2,940,758 | 924 | 2,185 | 36,505 | 893,508 | 35.1 |

**Table S4. 16S rRNA gene sequence similarity comparisons among JS1 genomes**

|  | B_OTU1 | AG183N14 | AG183J17 | AG183D10 | AG183P08 | AG183P14 | AG183P13 | AG183D19 | AG183B19 | AG183A05 | AG183M21 | AG183O15 | AG183G20 | AG183C13 | AG183F16 | AG183P18 | AG183A11 | AG183N04 | AG183P03 | SCGC_AD-561_N23 | ASPA | ASPC | ASLT | CDPL | CDPM | ASOZ | ASOY | TA biofilm JS1 MG bin |
| --- | --- | --- | --- | --- | --- | --- | --- | --- | --- | --- | --- | --- | --- | --- | --- | --- | --- | --- | --- | --- | --- | --- | --- | --- | --- | --- | --- | --- |
| AG183N14 | 99.8 | - |  |  |  |  |  |  |  |  |  |  |  |  |  |  |  |  |  |  |  |  |  |  |  |  |  |  |
| AG183J17 | 100 | 99.7 | - |  |  |  |  |  |  |  |  |  |  |  |  |  |  |  |  |  |  |  |  |  |  |  |  |  |
| AG183D10 | 99.8 | 100 | 99.4 | - |  |  |  |  |  |  |  |  |  |  |  |  |  |  |  |  |  |  |  |  |  |  |  |  |
| AG183P08 | 100 | 99.7 | 99.9 | 99.5 | - |  |  |  |  |  |  |  |  |  |  |  |  |  |  |  |  |  |  |  |  |  |  |  |
| AG183P14 | 99.8 | 99.9 | 99.5 | 99.8 | 99.6 | - |  |  |  |  |  |  |  |  |  |  |  |  |  |  |  |  |  |  |  |  |  |  |
| AG183P13 | 100 | 99.7 | 99.9 | 99.5 | 100 | 99.6 | - |  |  |  |  |  |  |  |  |  |  |  |  |  |  |  |  |  |  |  |  |  |
| AG183D19 | 99.8 | 99.9 | 99.7 | 100 | 99.7 | 99.9 | 99.7 | - |  |  |  |  |  |  |  |  |  |  |  |  |  |  |  |  |  |  |  |  |
| AG183B19 | 100 | 99.7 | 100 | 99.7 | 100 | 99.6 | 100 | 99.7 | - |  |  |  |  |  |  |  |  |  |  |  |  |  |  |  |  |  |  |  |
| AG183A05 | 99.8 | 100 | 99.7 | 100 | 99.7 | 99.9 | 99.7 | 100 | 99.7 | - |  |  |  |  |  |  |  |  |  |  |  |  |  |  |  |  |  |  |
| AG183M21 | 99.8 | 100 | 99.4 | 99.9 | 99.7 | 99.9 | 99.7 | 100 | 99.7 | 100 | - |  |  |  |  |  |  |  |  |  |  |  |  |  |  |  |  |  |
| AG183O15 | 99.8 | 100 | 99.4 | 100 | 99.5 | 99.8 | 99.5 | 100 | 99.7 | 100 | 99.9 | - |  |  |  |  |  |  |  |  |  |  |  |  |  |  |  |  |
| AG183G20 | 100 | 99.7 | 100 | 99.7 | 100 | 99.6 | 100 | 99.7 | 100 | 99.7 | 99.7 | 99.7 | - |  |  |  |  |  |  |  |  |  |  |  |  |  |  |  |
| AG183C13 | 100 | 99.7 | 100 | 99.7 | 100 | 99.6 | 100 | 99.7 | 100 | 99.7 | 99.7 | 99.7 | 100 | - |  |  |  |  |  |  |  |  |  |  |  |  |  |  |
| AG183F16 | 99.8 | 100 | 99.4 | 99.9 | 99.7 | 99.9 | 99.7 | 100 | 99.7 | 100 | 100 | 99.9 | 99.7 | 99.7 | - |  |  |  |  |  |  |  |  |  |  |  |  |  |
| AG183P18 | 99.8 | 99.6 | 99.8 | 99.6 | 99.8 | 99.5 | 99.8 | 99.5 | 99.8 | 99.6 | 99.6 | 99.6 | 99.8 | 99.8 | 99.6 | - |  |  |  |  |  |  |  |  |  |  |  |  |
| AG183A11 | 99.8 | 100 | 99.7 | 100 | 99.7 | 99.9 | 99.7 | 100 | 99.7 | 100 | 100 | 100 | 99.7 | 99.7 | 100 | 99.6 | - |  |  |  |  |  |  |  |  |  |  |  |
| AG183N04 | 99.8 | 100 | 99.7 | 100 | 99.7 | 99.9 | 99.7 | 100 | 99.7 | 100 | 100 | 100 | 99.7 | 99.7 | 100 | 99.6 | 100 | - |  |  |  |  |  |  |  |  |  |  |
| AG183P03 | 99.5 | 99.5 | 99.6 | 99.4 | 99.7 | 99.5 | 99.7 | 99.5 | 99.7 | 99.5 | 99.5 | 99.4 | 99.7 | 99.7 | 99.5 | 99.9 | 99.5 | 99.5 | - |  |  |  |  |  |  |  |  |  |
| SCGC_AD-561_N23 | 96.2 | 96.4 | 97 | 97 | 97 | 97 | 97 | 96.1 | 97.1 | 97 | 97 | 97 | 96.9 | 97 | 97 | 96.3 | 97 | 97.1 | 97 | - |  |  |  |  |  |  |  |  |
| ASPA01 | 96.6 | 93.2 | 93.5 | 93.3 | 93.5 | 93.3 | 93.5 | 93.2 | 93.9 | 93.6 | 93.3 | 93.3 | 93.8 | 93.8 | 93.3 | 93.5 | 93.8 | 93.9 | 93.3 | 97.7 | - |  |  |  |  |  |  |  |
| ASPC01 | 96.6 | 93.2 | 93.5 | 93.3 | 93.5 | 93.3 | 93.5 | 93.2 | 93.9 | 93.6 | 93.3 | 93.3 | 93.8 | 93.8 | 93.3 | 93.5 | 93.8 | 93.9 | 93.3 | 97.6 | 100 | - |  |  |  |  |  |  |
| ASLT01 | 96.4 | 93.1 | 93.5 | 93.4 | 93.5 | 93.3 | 93.5 | 93.1 | 93.8 | 93.5 | 93.4 | 93.4 | 93.6 | 93.7 | 93.4 | 93.3 | 93.7 | 93.8 | 93.3 | 97.6 | 99.9 | 99.9 | - |  |  |  |  |  |
| CDPL01 | 92.3 | 88 | 88 | 88.3 | 88.5 | 88.3 | 88.5 | 88 | 88.7 | 88.4 | 88.3 | 88.3 | 88.6 | 88.6 | 88.3 | 88.2 | 88.6 | 88.7 | 88.2 | 88.2 | 89.5 | 89.5 | 89.3 | - |  |  |  |  |
| CDPM01 | 96.6 | 93.1 | 93.3 | 93.2 | 93.4 | 93.2 | 93.4 | 93.1 | 93.8 | 93.5 | 93.2 | 93.2 | 93.6 | 93.7 | 93.2 | 93.3 | 93.7 | 93.8 | 93.1 | 93.1 | 99.6 | 99.6 | 99.5 | 89.3 | - |  |  |  |
| ASOZ01 | 91.8 | 87.9 | 88.3 | 88.2 | 88.3 | 88.1 | 88.3 | 87.9 | 88.6 | 88.3 | 88.2 | 88.2 | 88.4 | 88.5 | 88.2 | 88.1 | 88.5 | 88.6 | 88.1 | 88.1 | 89.5 | 89.5 | 89.3 | 99.6 | 89.3 | - |  |  |
| ASOY01 | 88.4 | 91.6 | 91.8 | 91.9 | 91.8 | 91.8 | 91.8 | 91.4 | 91.9 | 91.9 | 91.9 | 91.9 | 91.8 | 91.8 | 91.9 | 91.4 | 91.9 | 91.9 | 91.7 | 90.8 | 90.3 | 90.3 | 90.2 | 86 | 90.3 | 86 | - |  |
| TA biofilm JS1 MG bin | 88.2 | 91.1 | 91 | 91.1 | 91.1 | 91.1 | 91.1 | 91 | 91.1 | 91.2 | 91.1 | 91.1 | 91.2 | 91.1 | 91.1 | 91.2 | 91.2 | 91.2 | 91 | 90.4 | 92.4 | 92.4 | 92.2 | 89.8 | 92.4 | 89.8 | 100 | - |

**Table S5. Average nucleotide index comparisons among JS1 genomes**

|  | AG183A05 | AG183A11 | AG183B19 | AG183C13 | AG183D10 | AG183D19 | AG183F16 | AG183G20 | AG183J17 | AG183M21 | AG183N04 | AG183N14 | AG183O15 | AG183P03 | AG183P08 | AG183P13 | AG183P14 | AG183P18 | SCGC_AD  -561_N23 | ASLT01 | ASOY01 | ASOZ01 | ASPA01 | ASPC01 | CDPL01 | CDPM01 |
| --- | --- | --- | --- | --- | --- | --- | --- | --- | --- | --- | --- | --- | --- | --- | --- | --- | --- | --- | --- | --- | --- | --- | --- | --- | --- | --- |
| AG183A05 |  | 99.5 | 99.8 | 99.5 | 99.6 | 99.7 | 99.6 | 99.8 | 99.4 | 99.4 | 99.6 | 99.6 | 99.7 | 98.6 | 99.7 | 99.4 | 99.9 | 98.2 | 87.5 | 89.0 | 91.3 | 90.8 | 88.8 | 88.6 | 89.8 | 89.1 |
| AG183A11 | 99.5 |  | 99.5 | 99.3 | 99.1 | 99.5 | 99.4 | 99.6 | 99.4 | 99.4 | 99.2 | 99.2 | 99.5 | 98.0 | 99.6 | 99.4 | 99.4 | **97.8** | 87.6 | 89.3 | 87.3 | 90.8 | 89.3 | 88.9 | 91.4 | 89.1 |
| AG183B19 | 99.8 | 99.5 |  | 99.3 | 99.6 | 99.7 | 99.5 | 99.8 | 99.5 | 99.6 | 99.5 | 99.3 | 99.6 | **97.6** | 99.7 | 99.4 | 99.7 | **98.0** | 88.2 | 89.0 | 87.5 | 86.4 | 89.1 | 89.0 | 86.9 | 88.5 |
| AG183C13 | 99.5 | 99.3 | 99.3 |  | 99.0 | 99.5 | 99.3 | 99.6 | 99.2 | 99.3 | 99.2 | 99.2 | 99.5 | **97.9** | 99.5 | 99.2 | 99.4 | **96.8** | 87.8 | 89.2 | 89.0 | 90.2 | 88.7 | 88.6 | 89.1 | 89.1 |
| AG183D10 | 99.6 | 99.1 | 99.6 | 99.1 |  | 99.7 | 99.3 | 99.6 | 98.2 | 99.4 | 99.3 | 99.3 | 99.3 | 98.1 | 99.7 | 99.5 | 99.5 | 98.2 | 84.5 | 89.3 | 92.0 | 93.9 | 89.5 | 88.7 | 87.2 | 88.8 |
| AG183D19 | 99.7 | 99.5 | 99.7 | 99.5 | 99.7 |  | 99.2 | 99.8 | 99.3 | 99.7 | 99.4 | 99.5 | 99.6 | 98.5 | 99.7 | 99.8 | 99.8 | 98.3 | 86.9 | 88.6 | 87.7 | 0.0 | 88.4 | 88.4 | 84.6 | 89.0 |
| AG183F16 | 99.6 | 99.4 | 99.5 | 99.3 | 99.3 | 99.2 |  | 99.7 | 99.2 | 99.4 | 99.4 | 99.3 | 99.6 | **97.7** | 99.7 | 99.3 | 99.6 | **97.2** | 87.4 | 91.2 | 88.5 | 90.3 | 89.2 | 89.2 | 87.8 | 89.3 |
| AG183G20 | 99.8 | 99.6 | 99.8 | 99.6 | 99.6 | 99.8 | 99.7 |  | 99.5 | 99.5 | 99.5 | 99.5 | 99.6 | 98.5 | 99.8 | 99.6 | 99.8 | 98.0 | 87.2 | 90.5 | 88.8 | 92.4 | 88.7 | 88.4 | 92.0 | 88.9 |
| AG183J17 | 99.4 | 99.4 | 99.5 | 99.2 | 98.4 | 99.3 | 99.2 | 99.5 |  | 99.4 | 99.0 | 99.0 | 99.4 | 98.6 | 99.5 | 99.5 | 99.6 | 98.4 | 87.2 | 88.8 | 92.0 | 92.6 | 90.0 | 89.6 | 90.6 | 89.7 |
| AG183M21 | 99.4 | 99.4 | 99.6 | 99.3 | 99.4 | 99.7 | 99.4 | 99.5 | 99.4 |  | 99.3 | 99.4 | 99.5 | **97.8** | 99.7 | 99.3 | 99.3 | **97.6** | 88.1 | 90.1 | 88.4 | 88.0 | 88.7 | 87.9 | 85.4 | 88.8 |
| AG183N04 | 99.6 | 99.2 | 99.5 | 99.2 | 99.3 | 99.4 | 99.4 | 99.5 | 99.0 | 99.3 |  | 99.8 | 99.4 | 98.2 | 99.6 | 99.2 | 99.5 | **97.9** | 87.7 | 90.0 | 88.9 | 88.3 | 88.8 | 88.6 | 87.4 | 89.3 |
| AG183N14 | 99.6 | 99.2 | 99.3 | 99.2 | 99.3 | 99.5 | 99.3 | 99.5 | 99.0 | 99.4 | 99.8 |  | 99.4 | 98.2 | 99.6 | 99.2 | 99.5 | **97.9** | 88.5 | 88.7 | 89.7 | 90.7 | 89.2 | 88.7 | 87.7 | 89.3 |
| AG183O15 | 99.7 | 99.5 | 99.6 | 99.5 | 99.3 | 99.6 | 99.6 | 99.6 | 99.4 | 99.5 | 99.4 | 99.4 |  | **97.8** | 99.5 | 99.6 | 99.6 | **97.8** | 87.8 | 88.1 | 89.1 | 91.5 | 88.9 | 88.3 | 88.9 | 89.0 |
| AG183P03 | 98.6 | 98.0 | **97.7** | **97.9** | 98.1 | 98.5 | **97.7** | 98.5 | 98.6 | **97.7** | 98.2 | 98.2 | **97.9** |  | 98.8 | 98.4 | 98.5 | 98.7 | 87.3 | 89.3 | 87.8 | 91.4 | 88.7 | 88.6 | 88.3 | 89.0 |
| AG183P08 | 99.8 | 99.6 | 99.7 | 99.5 | 99.7 | 99.7 | 99.7 | 99.8 | 99.5 | 99.7 | 99.6 | 99.6 | 99.5 | 98.8 |  | 99.7 | 99.8 | 98.5 | 86.8 | 88.2 | 89.5 | 92.6 | 88.5 | 88.2 | 87.6 | 89.3 |
| AG183P13 | 99.4 | 99.4 | 99.4 | 99.2 | 99.5 | 99.8 | 99.3 | 99.6 | 99.5 | 99.3 | 99.2 | 99.1 | 99.6 | 98.4 | 99.7 |  | 99.3 | **97.4** | 88.1 | 89.5 | 90.3 | 91.9 | 90.1 | 89.0 | 88.1 | 89.5 |
| AG183P14 | 99.9 | 99.4 | 99.7 | 99.4 | 99.5 | 99.8 | 99.6 | 99.8 | 99.6 | 99.3 | 99.5 | 99.5 | 99.6 | 98.5 | 99.8 | 99.3 |  | **97.6** | 87.7 | 89.0 | 90.1 | 90.2 | 88.6 | 87.8 | 89.9 | 88.7 |
| AG183P18 | 98.2 | **97.8** | **98.0** | **96.8** | 98.2 | 98.3 | **97.2** | 98.0 | 98.4 | **97.6** | **97.9** | **97.9** | **97.8** | 98.7 | 98.5 | **97.4** | **97.7** |  | 87.5 | 87.3 | 84.3 | 87.3 | 88.6 | 88.4 | 84.9 | 88.8 |
| SCGC_AD-561_N23 | 87.5 | 87.6 | 88.2 | 87.8 | 84.5 | 86.9 | 87.4 | 87.2 | 87.2 | 88.1 | 87.7 | 88.5 | 87.8 | 87.3 | 86.8 | 88.1 | 87.7 | 87.5 |  | 87.9 | 90.7 | 89.0 | 89.2 | 88.2 | 81.9 | 89.5 |
| ASLT01 | 89.0 | 89.3 | 89.0 | 89.2 | 89.3 | 88.6 | 91.2 | 90.5 | 88.8 | 90.1 | 90.0 | 88.7 | 88.1 | 89.3 | 88.2 | 89.5 | 89.0 | 87.3 | 87.9 |  | 92.5 | 87.8 | 96.9 | 97.5 | 92.4 | 93.5 |
| ASOY01 | 91.3 | 87.3 | 87.5 | 89.0 | 92.0 | 87.7 | 88.5 | 88.8 | 92.0 | 88.4 | 88.9 | 89.7 | 89.1 | 87.8 | 89.5 | 90.3 | 90.1 | 84.3 | 90.7 | 92.5 |  | 90.3 | 93.6 | 95.4 | 90.4 | 95.8 |
| ASOZ01 | 90.8 | 90.8 | 86.4 | 90.2 | 93.9 | 0.0 | 90.3 | 92.4 | 92.6 | 88.0 | 88.3 | 90.7 | 91.5 | 91.4 | 92.6 | 91.9 | 90.2 | 87.3 | 89.0 | 87.8 | 90.3 |  | 88.5 | 89.5 | 93.0 | 87.5 |
| ASPA01 | 88.8 | 89.3 | 89.1 | 88.7 | 89.5 | 88.4 | 89.2 | 88.7 | 90.0 | 88.7 | 88.8 | 89.2 | 88.9 | 88.7 | 88.5 | 90.1 | 88.6 | 88.6 | 89.2 | 96.9 | 93.6 | 88.5 |  | 94.6 | 87.7 | 93.1 |
| ASPC01 | 88.6 | 88.9 | 89.0 | 88.6 | 88.7 | 88.4 | 89.2 | 88.4 | 89.6 | 87.9 | 88.6 | 88.7 | 88.3 | 88.6 | 88.2 | 89.0 | 87.8 | 88.4 | 88.2 | 97.5 | 95.4 | 89.5 | 94.6 |  | 87.5 | 92.7 |
| CDPL01 | 89.8 | 91.4 | 86.9 | 89.1 | 87.2 | 84.6 | 87.8 | 92.0 | 90.6 | 85.4 | 87.4 | 87.7 | 88.9 | 88.3 | 87.6 | 88.1 | 89.9 | 84.9 | 81.9 | 92.4 | 90.4 | 93.0 | 87.7 | 87.5 |  | 89.3 |
| CDPM01 | 89.1 | 89.1 | 88.5 | 89.1 | 88.8 | 89.0 | 89.3 | 88.9 | 89.7 | 88.8 | 89.3 | 89.3 | 89.0 | 89.0 | 89.3 | 89.5 | 88.7 | 88.8 | 89.5 | 93.5 | 95.8 | 87.5 | 93.2 | 92.7 | 89.3 |  |

**Table S6. Conserved single copy gene sets**

|  | PFAM ID | HMM name | HMM cutoff | Description of bacterial genes | RS-JS1 cSAG Protein ID in RAST |
| --- | --- | --- | --- | --- | --- |
| 1 | PF03485 | Arg_tRNA_synt_N | 32.55 | Arginyl tRNA synthetase N terminal domain |  |
| 2 | PF03484 | B5 | 26.55 | tRNA synthetase B5 domain | fig\|6666666.379707.peg.523 |
| 3 | PF01121 | CoaE | 84.65 | Dephospho‐CoA kinase | fig\|6666666.379707.peg.690 |
| 4 | PF03772 | Competence | 82.7 | Competence protein | fig\|6666666.379707.peg.2096 |
| 5 | PF03602 | Cons_hypoth95 | 38.8 | Conserved hypothetical protein 95 | fig\|6666666.379707.peg.510 |
| 6 | PF06418 | CTP_synth_N | 208 | CTP synthase N‐terminus | fig\|6666666.379707.peg.1958 |
| 7 | PF02224 | Cytidylate_kin | 79.35 | Cytidylate kinase | fig\|6666666.379707.peg.506 |
| 8 | PF00712 | DNA_pol3_beta | 48.7 | DNA polymerase III beta subunit, N‐terminal domain |  |
| 9 | PF02767 | DNA_pol3_beta_2 | 49.65 | DNA polymerase III beta subunit, central domain |  |
| 10 | PF02768 | DNA_pol3_beta_3 | 44.5 | DNA polymerase III beta subunit, C‐terminal domain |  |
| 11 | PF00035 | dsrm | 20.2 | Double‐stranded RNA binding motif | fig\|6666666.379707.peg.531 |
| 12 | PF00889 | EF_TS | 108.35 | Elongation factor TS | fig\|6666666.379707.peg.536 |
| 13 | PF01176 | eIF-1a | 39.25 | Translation initiation factor 1A / IF‐1 | fig\|6666666.379707.peg.1055 |
| 14 | PF00113 | Enolase_C | 206.35 | Enolase, C‐terminal TIM barrel domain | fig\|6666666.379707.peg.1074 |
| 15 | PF03952 | Enolase_N | 92.45 | 45 Enolase, N‐terminal domain | fig\|6666666.379707.peg.1074 |
| 16 | PF06574 | FAD_syn | 73.25 | 25 FAD synthetase |  |
| 17 | PF03147 | FDX-ACB | 39.75 | Ferredoxin‐fold anticodon binding domain | fig\|6666666.379707.peg.523 |
| 18 | PF01687 | Flavokinase | 59.3 | Riboflavin kinase |  |
| 19 | PF02938 | GAD | 40.05 | GAD domain | fig\|6666666.379707.peg.1687 |
| 20 | PF02527 | GidB | 77.85 | rRNA small subunit methyltransferase G | fig\|6666666.379707.peg.1483 |
| 21 | PF00958 | GMP_synt_C | 61.3 | GMP synthase C terminal domain |  |
| 22 | PF01025 | GrpE | 72.25 | GrpE | fig\|6666666.379707.peg.1993 |
| 23 | PF01018 | GTP1_OBG | 98.9 | GTP1/OBG | fig\|6666666.379707.peg.683 |
| 24 | PF11987 | IF?2 | 61.7 | Translation‐initiation factor 2 | fig\|6666666.379707.peg.1521 |
| 25 | PF04760 | IF2_N | 29.95 | Translation initiation factor IF‐2, N‐terminal region | fig\|6666666.379707.peg.550 |
| 26 | PF00707 | IF3_C | 51.55 | Translation initiation factor IF‐3, C‐terminal domain | fig\|6666666.379707.peg.784 |
| 27 | PF05198 | IF3_N | 47.95 | Translation initiation factor IF‐3, N‐terminal domain |  |
| 28 | PF01715 | IPPT | 129.15 | IPP transferase | fig\|6666666.379707.peg.1467 |
| 29 | PF06421 | LepA_C | 79.7 | GTP‐binding protein LepA C‐terminus | fig\|6666666.379707.peg.2084 |
| 30 | PF01795 | Methyltransf_5 | 174.05 | MraW methylase family |  |
| 31 | PF02873 | MurB_C | 46.15 | UDP‐N‐acetylenolpyruvoylglucosamine reductase, C‐terminal domain | fig\|6666666.379707.peg.2 |
| 32 | PF08529 | NusA_N | 61.55 | NusA N‐terminal domain | fig\|6666666.379707.peg.547 |
| 33 | PF02410 | Oligomerisation | 44.9 | Oligomerisation domain | fig\|6666666.379707.peg.1481 |
| 34 | PF01195 | Pept_tRNA_hydro | 99.15 | Peptidyl‐tRNA hydrolase | fig\|6666666.379707.peg.473 |
| 35 | PF01252 | Peptidase_A8 | 61.35 | Signal peptidase (SPase) II | fig\|6666666.379707.peg.8 |
| 36 | PF00162 | PGK | 236 | Phosphoglycerate kinase | fig\|6666666.379707.peg.1819 |
| 37 | PF02912 | Phe_tRNA-synt_N | 33.55 | Aminoacyl tRNA synthetase class II, N‐terminal domain | fig\|6666666.379707.peg.920 |
| 38 | PF03726 | PNPase | 25.6 | Polyribonucleotide nucleotidyltransferase, RNA binding domain | fig\|6666666.379707.peg.2416 |
| 39 | PF01416 | PseudoU_synth_1 | 42.45 | tRNA pseudouridine synthase | fig\|6666666.379707.peg.2316 |
| 40 | PF02033 | RBFA | 44.9 | Ribosome‐binding factor A | fig\|6666666.379707.peg.1519 |
| 41 | PF00154 | RecA | 276.55 | recA bacterial DNA recombination protein | fig\|6666666.379707.peg.1952 |
| 42 | PF02132 | RecR | 21.25 | RecR protein | fig\|6666666.379707.peg.1788 |
| 43 | PF00825 | Ribonuclease_P | 40.55 | Ribonuclease P |  |
| 44 | PF00687 | Ribosomal_L1 | 75.1 | Ribosomal protein L1p/L10e family | fig\|6666666.379707.peg.1386 |
| 45 | PF00466 | Ribosomal_L10 | 37.3 | Ribosomal protein L10 | fig\|6666666.379707.peg.1385 |
| 46 | PF00298 | Ribosomal_L11 | 42.65 | Ribosomal protein L11, RNA binding domain | fig\|6666666.379707.peg.1387 |
| 47 | PF03946 | Ribosomal_L11_N | 45.9 | Ribosomal protein L11, N‐terminal domain | fig\|6666666.379707.peg.1387 |
| 48 | PF00542 | Ribosomal_L12 | 44 | Ribosomal protein L7/L12 C‐terminal domain | fig\|6666666.379707.peg.1384 |
| 49 | PF00572 | Ribosomal_L13 | 81.3 | Ribosomal protein L13 | fig\|6666666.379707.peg.2570 |
| 50 | PF00238 | Ribosomal_L14 | 79.55 | Ribosomal protein L14p/L23e | fig\|6666666.379707.peg.2402 |
| 51 | PF00252 | Ribosomal_L16 | 77.45 | Ribosomal protein L16p/L10e | fig\|6666666.379707.peg.2406 |
| 52 | PF01196 | Ribosomal_L17 | 55.75 | Ribosomal protein L17 | fig\|6666666.379707.peg.1061 |
| 53 | PF00828 | Ribosomal_L18e | 37.55 | Ribosomal protein L18e/L15 | fig\|6666666.379707.peg.1051 |
| 54 | PF00861 | Ribosomal_L18p | 54.85 | Ribosomal L18p/L5e family | fig\|6666666.379707.peg.1838 |
| 55 | PF01245 | Ribosomal_L19 | 71.7 | Ribosomal protein L19 |  |
| 56 | PF00181 | Ribosomal_L2 | 52.45 | Ribosomal Proteins L2, RNA binding domain | fig\|6666666.379707.peg.1810 |
| 57 | PF03947 | Ribosomal_L2_C | 87.3 | Ribosomal Proteins L2, C‐terminal domain | fig\|6666666.379707.peg.1810 |
| 58 | PF00453 | Ribosomal_L20 | 70.5 | Ribosomal protein L20 | fig\|6666666.379707.peg.782 |
| 59 | PF00829 | Ribosomal_L21p | 53.45 | Ribosomal prokaryotic L21 protein | fig\|6666666.379707.peg.1090 |
| 60 | PF00237 | Ribosomal_L22 | 57.6 | Ribosomal protein L22p/L17e | fig\|6666666.379707.peg.1812 |
| 61 | PF00276 | Ribosomal_L23 | 37.45 | Ribosomal protein L23 | fig\|6666666.379707.peg.1809 |
| 62 | PF01016 | Ribosomal_L27 | 55.4 | Ribosomal L27 protein | fig\|6666666.379707.peg.1091 |
| 63 | PF00830 | Ribosomal_L28 | 36.8 | Ribosomal L28 family | fig\|6666666.379707.peg.508 |
| 64 | PF00831 | Ribosomal_L29 | 33.7 | Ribosomal L29 protein | fig\|6666666.379707.peg.2405 |
| 65 | PF00297 | Ribosomal_L3 | 66.35 | Ribosomal protein L3 | fig\|6666666.379707.peg.1807 |
| 66 | PF01783 | Ribosomal_L32p | 28.2 | Ribosomal L32p protein family |  |
| 67 | PF01632 | Ribosomal_L35p | 29.5 | Ribosomal protein L35 | fig\|6666666.379707.peg.783 |
| 68 | PF00573 | Ribosomal_L4 | 99.95 | Ribosomal protein L4/L1 family | fig\|6666666.379707.peg.1808 |
| 69 | PF00281 | Ribosomal_L5 | 34.55 | Ribosomal protein L5 | fig\|6666666.379707.peg.1839 |
| 70 | PF00673 | Ribosomal_L5_C | 61.25 | ribosomal L5P family C‐terminus | fig\|6666666.379707.peg.1839 |
| 71 | PF00347 | Ribosomal_L6 | 53.25 | Ribosomal protein L6 | fig\|6666666.379707.peg.1837 |
| 72 | PF03948 | Ribosomal_L9_C | 37.95 | Ribosomal protein L9, C‐terminal domain | fig\|6666666.379707.peg.173 |
| 73 | PF01281 | Ribosomal_L9_N | 30.55 | Ribosomal protein L9, N‐terminal domain | fig\|6666666.379707.peg.173 |
| 74 | PF00338 | Ribosomal_S10 | 58.05 | Ribosomal protein S10p/S20e | fig\|6666666.379707.peg.1806 |
| 75 | PF00411 | Ribosomal_S11 | 73.45 | Ribosomal protein S11 | fig\|6666666.379707.peg.1058 |
| 76 | PF00164 | Ribosomal_S12 | 84.3 | Ribosomal protein S12 | fig\|6666666.379707.peg.1292 |
| 77 | PF00416 | Ribosomal_S13 | 56.2 | Ribosomal protein S13/S18 | fig\|6666666.379707.peg.1057 |
| 78 | PF00312 | Ribosomal_S15 | 41.7 | Ribosomal protein S15 |  |
| 79 | PF00886 | Ribosomal_S16 | 36.25 | Ribosomal protein S16 |  |
| 80 | PF00366 | Ribosomal_S17 | 41.3 | Ribosomal protein S17 | fig\|6666666.379707.peg.2404 |
| 81 | PF01084 | Ribosomal_S18 | 36.3 | Ribosomal protein S18 |  |
| 82 | PF00203 | Ribosomal_S19 | 54.9 | Ribosomal protein S19 | fig\|6666666.379707.peg.1811 |
| 83 | PF00318 | Ribosomal_S2 | 133.15 | Ribosomal protein S2 | fig\|6666666.379707.peg.537 |
| 84 | PF01649 | Ribosomal_S20p | 35.95 | Ribosomal protein S20 | fig\|6666666.379707.peg.2094 |
| 85 | PF00189 | Ribosomal_S3_C | 49.5 | Ribosomal protein S3, C‐terminal domain | fig\|6666666.379707.peg.2407 |
| 86 | PF00163 | Ribosomal_S4 | 32.9 | Ribosomal protein S4/S9 N‐terminal domain | fig\|6666666.379707.peg.1059 |
| 87 | PF00333 | Ribosomal_S5 | 42.55 | Ribosomal protein S5, N‐terminal domain | fig\|6666666.379707.peg.1050 |
| 88 | PF03719 | Ribosomal_S5_C | 44.9 | Ribosomal protein S5, C‐terminal domain | fig\|6666666.379707.peg.1050 |
| 89 | PF01250 | Ribosomal_S6 | 45.2 | Ribosomal protein S6 | fig\|6666666.379707.peg.1213 |
| 90 | PF00177 | Ribosomal_S7 | 95.55 | Ribosomal protein S7p/S5e | fig\|6666666.379707.peg.1291 |
| 91 | PF00410 | Ribosomal_S8 | 72.35 | Ribosomal protein S8 | fig\|6666666.379707.peg.1764 |
| 92 | PF00380 | Ribosomal_S9 | 70.4 | Ribosomal protein S9/S16 | fig\|6666666.379707.peg.2571 |
| 93 | PF01782 | RimM | 30.65 | RimM N‐terminal domain |  |
| 94 | PF01000 | RNA_pol_A_bac | 34.3 | RNA polymerase Rpb3/RpoA insert domain | fig\|6666666.379707.peg.1060 |
| 95 | PF03118 | RNA_pol_A_CTD | 38.9 | Bacterial RNA polymerase, alpha chain C terminal domain | fig\|6666666.379707.peg.1060 |
| 96 | PF01193 | RNA_pol_L | 34.35 | RNA polymerase Rpb3/Rpb11 dimerization domain | fig\|6666666.379707.peg.1060 |
| 97 | PF04997 | RNA_pol_Rpb1_1 | 157.25 | RNA polymerase Rpb1, domain 1 |  |
| 98 | PF00623 | RNA_pol_Rpb1_2 | 79.8 | RNA polymerase Rpb1, domain 2 |  |
| 99 | PF04983 | RNA_pol_Rpb1_3 | 41.85 | RNA polymerase Rpb1, domain 3 |  |
| 100 | PF05000 | RNA_pol_Rpb1_4 | 24.6 | RNA polymerase Rpb1, domain 4 |  |
| 101 | PF04998 | RNA_pol_Rpb1_5 | 119.4 | RNA polymerase Rpb1, domain 5 | fig\|6666666.379707.peg.1293 |
| 102 | PF04563 | RNA_pol_Rpb2_1 | 39.95 | RNA polymerase beta subunit | fig\|6666666.379707.peg.1383 |
| 103 | PF04561 | RNA_pol_Rpb2_2 | 37.15 | RNA polymerase Rpb2, domain 2 | fig\|6666666.379707.peg.1383 |
| 104 | PF04565 | RNA_pol_Rpb2_3 | 45.6 | RNA polymerase Rpb2, domain 3 | fig\|6666666.379707.peg.1383 |
| 105 | PF10385 | RNA_pol_Rpb2_45 | 36.6 | RNA polymerase beta subunit external 1 domain | fig\|6666666.379707.peg.394 |
| 106 | PF00562 | RNA_pol_Rpb2_6 | 258.45 | RNA polymerase Rpb2, domain 6 | fig\|6666666.379707.peg.394 |
| 107 | PF04560 | RNA_pol_Rpb2_7 | 46.45 | RNA polymerase Rpb2, domain 7 | fig\|6666666.379707.peg.394 |
| 108 | PF01765 | RRF | 101.65 | Ribosome recycling factor |  |
| 109 | PF07499 | RuvA_C | 16.6 | RuvA, C‐terminal domain | fig\|6666666.379707.peg.1875 |
| 110 | PF01330 | RuvA_N | 26.85 | RuvA N terminal domain | fig\|6666666.379707.peg.1875 |
| 111 | PF05491 | RuvB_C | 49.5 | Holliday junction DNA helicase ruvB C‐terminus | fig\|6666666.379707.peg.1893 |
| 112 | PF02773 | S-AdoMet_synt_C | 108.9 | S‐adenosylmethionine synthetase, C‐terminal domain | fig\|6666666.379707.peg.1008 |
| 113 | PF02772 | S-AdoMet_synt_M | 77.05 | S‐adenosylmethionine synthetase, central domain | fig\|6666666.379707.peg.1008 |
| 114 | PF00584 | SecE | 27.5 | SecE/Sec61‐gamma subunits of protein translocation complex | fig\|6666666.379707.peg.1890 |
| 115 | PF03840 | SecG | 29.2 | Preprotein translocase SecG subunit | fig\|6666666.379707.peg.1313 |
| 116 | PF00344 | SecY | 180.7 | SecY translocase | fig\|6666666.379707.peg.1052 |
| 117 | PF02403 | Seryl_tRNA_N | 43.15 | Seryl‐tRNA synthetase N‐terminal domain |  |
| 118 | PF01668 | SmpB | 43.4 | SmpB protein | fig\|6666666.379707.peg.1263 |
| 119 | PF02978 | SRP_SPB | 52.8 | Signal peptide binding domain |  |
| 120 | PF00763 | THF_DHG_CYH | 62.2 | Tetrahydrofolate dehydrogenase/cyclohydrolase, catalytic domain | fig\|6666666.379707.peg.1517 |
| 121 | PF02882 | THF_DHG_CYH_C | 103.7 | Tetrahydrofolate dehydrogenase/cyclohydrolase, NAD(P)‐binding domain | fig\|6666666.379707.peg.1517 |
| 122 | PF00121 | TIM | 140.2 | Triosephosphate isomerase | fig\|6666666.379707.peg.1312 |
| 123 | PF08275 | Toprim_N | 58.6 | DNA primase catalytic core, N‐terminal domain | fig\|6666666.379707.peg.682 |
| 124 | PF03461 | TRCF | 40.05 | TRCF domain | fig\|6666666.379707.peg.2399 |
| 125 | PF05698 | Trigger_C | 55.15 | Bacterial trigger factor protein (TF) C‐terminus | fig\|6666666.379707.peg.399 |
| 126 | PF05697 | Trigger_N | 61.8 | Bacterial trigger factor protein (TF) | fig\|6666666.379707.peg.399 |
| 127 | PF01746 | tRNA_m1G_MT | 80.75 | tRNA (Guanine‐1)‐methyltransferase |  |
| 128 | PF00750 | tRNA-synt_1d | 120.85 | tRNA synthetases class I (R) |  |
| 129 | PF01409 | tRNA-synt_2d | 161.15 | tRNA synthetases class II core domain (F) | fig\|6666666.379707.peg.920 |
| 130 | PF01509 | TruB_N | 83.55 | TruB family pseudouridylate synthase (N terminal domain) | fig\|6666666.379707.peg.2427 |
| 131 | PF00627 | UBA | 13.3 | UBA/TS‐N domain |  |
| 132 | PF02130 | UPF0054 | 59.35 | Uncharacterized protein family UPF0054 | fig\|6666666.379707.peg.1931 |
| 133 | PF02367 | UPF0079 | 46.1 | Uncharacterised P‐loop hydrolase UPF0079 | fig\|6666666.379707.peg.2151 |
| 134 | PF03652 | UPF0081 | 60.75 | Uncharacterised protein family (UPF0081) | fig\|6666666.379707.peg.761 |
| 135 | PF12344 | UvrB | 33.75 | Ultra‐violet resistance protein B | fig\|6666666.379707.peg.687 |
| 136 | PF08459 | UvrC_HhH_N | 81.9 | UvrC Helix‐hairpin‐helix N‐terminal | fig\|6666666.379707.peg.1118 |
| 137 | PF10458 | Val_tRNA-synt_C | 26.5 | Valyl tRNA synthetase tRNA binding arm | fig\|6666666.379707.peg.405 |
| 138 | PF06071 | YchF-GTPase_C | 61.9 | Protein of unknown function (DUF933) | fig\|6666666.379707.peg.1428 |
| 139 | PF06689 | zf-C4_ClpX | 31.7 | ClpX C4‐type zinc finger | fig\|6666666.379707.peg.402 |

**Table S7. Percent identity of ABC transporter for maltose and galactose among JS1 genomes**

| **Protein ID in RAST** | **Function** | **GHOSTZ result** | **Categories** | SL SAG co-assembly | SCGC AAA255-G05 | Aarhus Bay SAG I22 | SL MG bin | SCGC AB-164-G04 | SCGC AAA255-N14 | SCGC AAA255-E04 | SCGC AB-164-A22 | SCGC AD-561-N23 | TA biofilm SAG 231 | TA biofilm MG bin | JGI 0000014-F07 | Aarhus Bay SAG B17 | JGI 0000059-I14 |
| --- | --- | --- | --- | --- | --- | --- | --- | --- | --- | --- | --- | --- | --- | --- | --- | --- | --- |
| fig\|6666666.379707.peg.1600 | Predicted galactoside ABC transporter type II, sugar-binding protein | K17315 | Galactose transporters and metabolism | 27.65 | 0 | 27.02 | 0 | 0 | 27.65 | 0 | 24.37 | 0 | 29.62 | 0 | 22.35 | 23.55 | 24.25 |
| fig\|6666666.379707.peg.1601 | Predicted galactoside ABC transporter type II, permease protein 1 | K10118 | Galactose transporters and metabolism | 34.04 | 0 | 34.46 | 0 | 0 | 31.27 | 0 | 34.08 | 0 | 30.68 | 32.26 | 29.97 | 27.23 | 33.09 |
| fig\|6666666.379707.peg.1602 | Predicted galactoside ABC transporter type II, permease protein 2 | K10119 | Galactose transporters and metabolism | 31.14 | 0 | 32.96 | 0 | 0 | 38.19 | 0 | 30.94 | 0 | 40.62 | 34.6 | 31.32 | 29.63 | 32.96 |
| fig\|6666666.379707.peg.2266 | ABC sugar transporter, ATP-binding component | K02017 | Maltose transporters and metabolism | 42.31 | 42.49 | 42.24 | 33.33 | 33.33 | 45.42 | 42.49 | 40.08 | 0 | 42.36 | 38.61 | 41.84 | 38.82 | 42.31 |
| fig\|6666666.379707.peg.2265 | Maltose/maltodextrin transport ATP-binding protein MalK (EC 3.6.3.19) | K11072 | Maltose transporters and metabolism | 36.89 | 40.97 | 41.59 | 27.91 | 32.64 | 42 | 40.97 | 38.26 | 0 | 40.57 | 37.54 | 43.72 | 38.99 | 39.78 |
| fig\|6666666.379707.peg.2262 | Maltose/maltodextrin ABC transporter, permease protein MalG | K02026 | Maltose transporters and metabolism | 33.2 | 25.16 | 39.31 | 0 | 0 | 34.95 | 25.16 | 27.62 | 0 | 36.76 | 32.86 | 43.75 | 34.18 | 28.95 |
| fig\|6666666.379707.peg.2264 | ABC transport system, sugar-binding protein | K02027 | Maltose transporters and metabolism | 34.69 | 0 | 24.17 | 0 | 0 | 27.45 | 0 | 0 | 0 | 25.91 | 0 | 26.34 | 0 | 23.42 |
| fig\|6666666.379707.peg.2263 | Dihydroxyacetone ABC transport system, permease protein 2 # predicted | K02025 | Maltose transporters and metabolism | 34.27 | 0 | 35.25 | 0 | 0 | 33.21 | 0 | 28.74 | 0 | 31.05 | 40.23 | 33.73 | 29.7 | 33.64 |

**Table S8. Comparison of BMC gene loci between RS JS1-cSAG and the SL SAG co-assembly**

| NCBI contig name in the SL SAG co-assembly | SL SAG co-assembly Protein ID (RAST) | Gene | BMC component | Identities | RS JS1- cSCG Protein ID (RAST) | RS JS1- cSCG contig number |
| --- | --- | --- | --- | --- | --- | --- |
| gi\|482623737\|gb\|AQYX01000101.1\| | fig\|6666666.190053.peg.1785 | l-Rhamnose isomerase (EC 5.3.1.14) |  | 92.96 | fig\|6666666.379707.peg.2232 | 410 |
| gi\|482623737\|gb\|AQYX01000101.1\| | fig\|6666666.190053.peg.1786 | Hypothetical protein |  |  |  |  |
| gi\|482623737\|gb\|AQYX01000101.1\| | fig\|6666666.190053.peg.1787 | Ethanolamine utilization protein similar to PduA/PduJ | BMC shell protein | 97.2 | fig\|6666666.379707.peg.2236 | 410 |
| gi\|482623737\|gb\|AQYX01000101.1\| | fig\|6666666.190053.peg.1788 | Ethanolamine utilization polyhedral-body-like protein EutN | BMC shell protein | 95.83 | fig\|6666666.379707.peg.2235 | 410 |
| gi\|482623737\|gb\|AQYX01000101.1\| | fig\|6666666.190053.peg.1789 | Ethanolamine utilization polyhedral-body-like protein EutN | BMC shell protein | 91.58 | fig\|6666666.379707.peg.2234 | 410 |
| gi\|482623737\|gb\|AQYX01000101.1\| | fig\|6666666.190053.peg.1790 | Cob(III)alamin reductase @ Cob(II)alamin reductase |  | 94.52 | fig\|6666666.379707.peg.2233 | 410 |
| gi\|482623737\|gb\|AQYX01000101.1\| | fig\|6666666.190053.peg.1791 | Propanediol utilization polyhedral body protein PduT | BMC shell protein | 95.73 | fig\|6666666.379707.peg.2321 | 432 |
| gi\|482623737\|gb\|AQYX01000101.1\| | fig\|6666666.190053.peg.1792 | CoA-acylating propionaldehyde dehydrogenase |  | 91.01 | fig\|6666666.379707.peg.2336 | 439 |
| gi\|482623737\|gb\|AQYX01000101.1\| | fig\|6666666.190053.peg.1793 | Hypothetical protein |  |  |  |  |
| gi\|482623737\|gb\|AQYX01000101.1\| | fig\|6666666.190053.peg.1794 | Ethanolamine utilization polyhedral-body-like protein EutN | BMC shell protein | 95.83 | fig\|6666666.379707.peg.2259 | 418 |
| gi\|482623737\|gb\|AQYX01000101.1\| | fig\|6666666.190053.peg.1795 | Ethanolamine utilization polyhedral-body-like protein EutN | BMC shell protein | 91.86 | fig\|6666666.379707.peg.2258 | 418 |
| gi\|482623737\|gb\|AQYX01000101.1\| | fig\|6666666.190053.peg.1796 | Ribose 5-phosphate isomerase B (EC 5.3.1.6) |  | 94.54 | fig\|6666666.379707.peg.2257 | 418 |
| gi\|482623737\|gb\|AQYX01000101.1\| | fig\|6666666.190053.peg.1797 | Deoxyribose-phosphate aldolase (EC 4.1.2.4) |  | 90.77 | fig\|6666666.379707.peg.2041 | 341 |
| gi\|482623737\|gb\|AQYX01000101.1\| | fig\|6666666.190053.peg.1798 | Ethanolamine utilization protein similar to PduL |  | 98.2 | Partial sequence (800~1150) | 592 |

**Table S9. Diagnostic markers for the diderm cell envelope structure**

|  |  |  | **RS JS1-cSAG** |  | **SL SAG co-assembly** |  | **OP9** |  |
| --- | --- | --- | --- | --- | --- | --- | --- | --- |
| **Protein name** | **Function/annotation** | **Pfam number** | **Protein ID in RAST** | **e-value** | **Protein ID in RAST** | **e-value** | **Protein ID in RAST** | **e-value** |
| BamA(YaeT) | OM assembly | PF07244 | fig\|6666666.379707.peg.1804 | 0.000000015 | fig\|6666666.138344.peg.944 | 0.000000047 | fig\|6666666.140011.peg.618 | 6.90E-09 |
|  | OM assembly | PF01103 | fig\|6666666.379707.peg.1804 | 2.8E-50 | fig\|6666666.138344.peg.944 | 3.20E-50 | fig\|6666666.140011.peg.618 | 1.00E-45 |
| TolC | Type 1 secretion | PF02321 | fig\|6666666.379707.peg.295 | 0.0000019 |  |  | fig\|6666666.140011.peg.723 | 5.00E-14 |
| Secretin | Type 2, 3 secretion | PF00263 |  |  |  |  | fig\|6666666.140011.peg.1647 | 2.20E-38 |
| Secretin/TonB | Type 2, 3 secretion | PF07660 |  |  |  |  | fig\|6666666.140011.peg.1647 | 1.20E-07 |
| FlgH | Flagellar L-ring | PF02107 |  |  |  |  | fig\|6666666.140011.peg.1566 | 5.29E-31 |
| FlgI | Flagellar P-ring | PF02119 |  |  |  |  | fig\|6666666.140011.peg.1549 | 6.75E-80 |

**Table S10. Enzymes involved in the utilization of threonine and aspartate**

| **Category** | **ProteinID (RAST)** | **Gene** | **Reaction** |
| --- | --- | --- | --- |
| Threonine utilization and biosynthesis | fig\|6666666.379707.peg.1632 | L-threonine aldolase (EC 4.1.2.5) | L-Threonine <=> Glycine + Acetaldehyde |
|  | fig\|6666666.379707.peg.2045 | Serine hydroxymethyltransferase (EC 2.1.2.1) | 5,10-methylenetetrahydrofolate + glycine + H_2_O <=> tetrahydrofolate + L-serine |
|  | fig\|6666666.379707.peg.652 | L-threonine 3-dehydrogenase (EC 1.1.1.103) | L-threonine + NAD^+^ <=> L-2-amino-3-oxobutanoate + NADH + H^+^ |
| Aspartate utilization and biosynthesis | fig\|6666666.379707.peg.2517 | Adenylosuccinate synthetase (EC 6.3.4.4) | Guanosine 5'-triphosphate + Inosine 5'-monophosphate + L-aspartate <=> Guanosine 5'-diphosphate + phosphate + Adenylosuccinate |
|  | fig\|6666666.379707.peg.896 | Adenylosuccinate lyase (EC 4.3.2.2) | Adenylosuccinate <=> Fumarate + AMP |
|  | fig\|6666666.379707.peg.2066 | Fumarate hydratase class I (EC 4.2.1.2) | L-Malate <=> Fumarate + H_2_O |
|  | fig\|6666666.379707.peg.2351 | NAD-dependent malic enzyme (EC 1.1.1.38) | L-Malate + NAD^+^ <=> Pyruvate + CO_2_ + NADH + H^+^ |
|  | fig\|6666666.379707.peg.1615 | L-aspartate oxidase (EC 1.4.3.16) | L-Aspartate + Oxygen <=> Iminoaspartate + Hydrogen peroxide |
|  | fig\|6666666.379707.peg.628 | Oxaloacetate decarboxylase (EC 4.1.1.3) | Oxaloacetate <=> Pyruvate + CO_2_ |
