# Supplementary material for: Genomic Insight Into the Predominance of Candidate Phylum Atribacteria JS1 Lineage in Marine Sediments
Source: Front Microbiol. 2018 Nov 29;9:2909. doi: 10.3389/fmicb.2018.02909 (PMC6281690; doi:10.3389/fmicb.2018.02909)
Supplement: Supplementary file 2 [file Presentation_1.PPTX]

## Slide 1
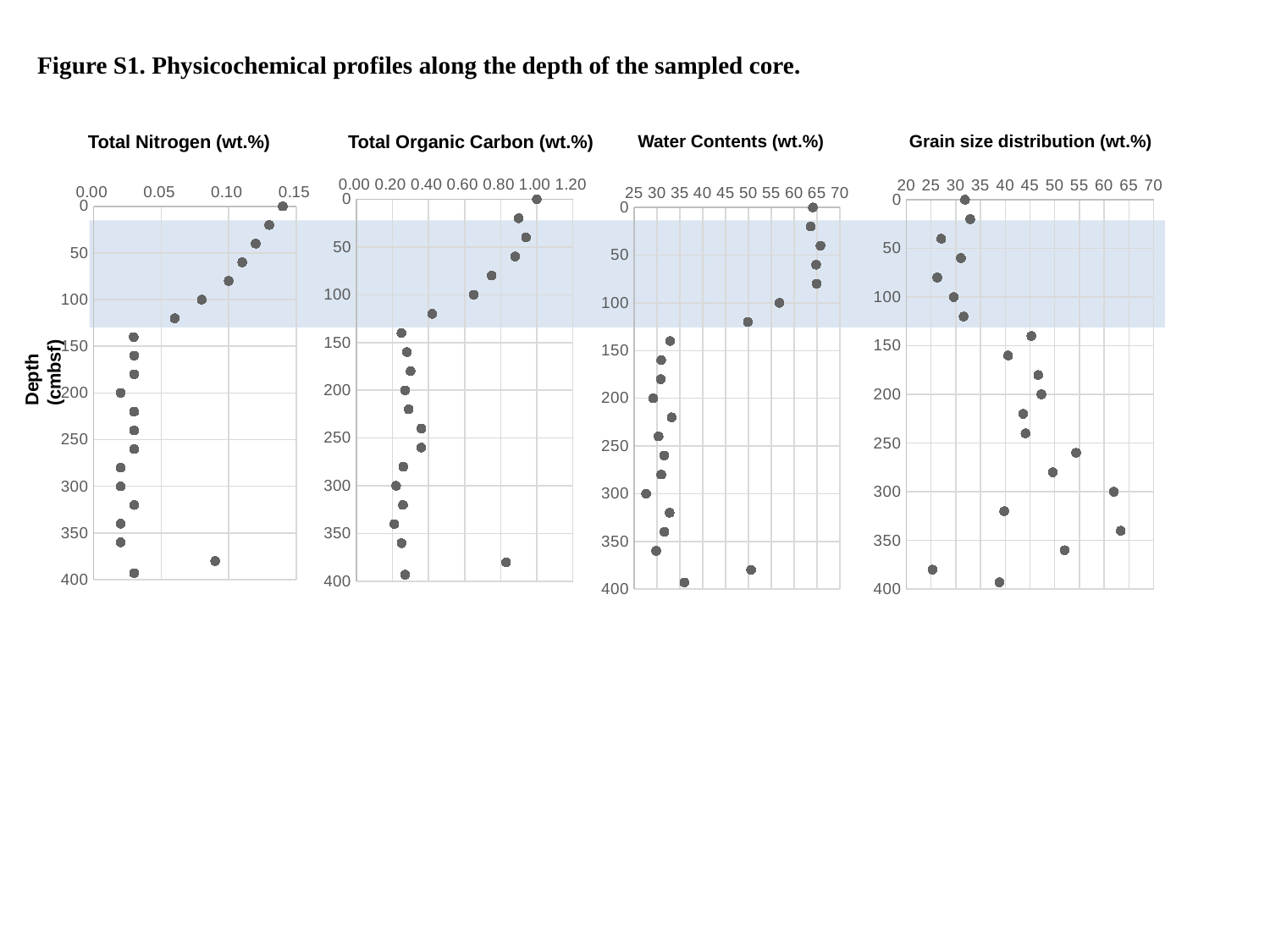

Figure S1. Physicochemical profiles along the depth of the sampled core.
### Chart
| Category | |
|---|---|Total Nitrogen (wt.%)
Total Organic Carbon (wt.%)
Water Contents (wt.%)
Grain size distribution (wt.%)
### Chart
| Category | |
|---|---|
### Chart
| Category | |
|---|---|
### Chart
| Category | |
|---|---|Depth (cmbsf)

## Slide 2
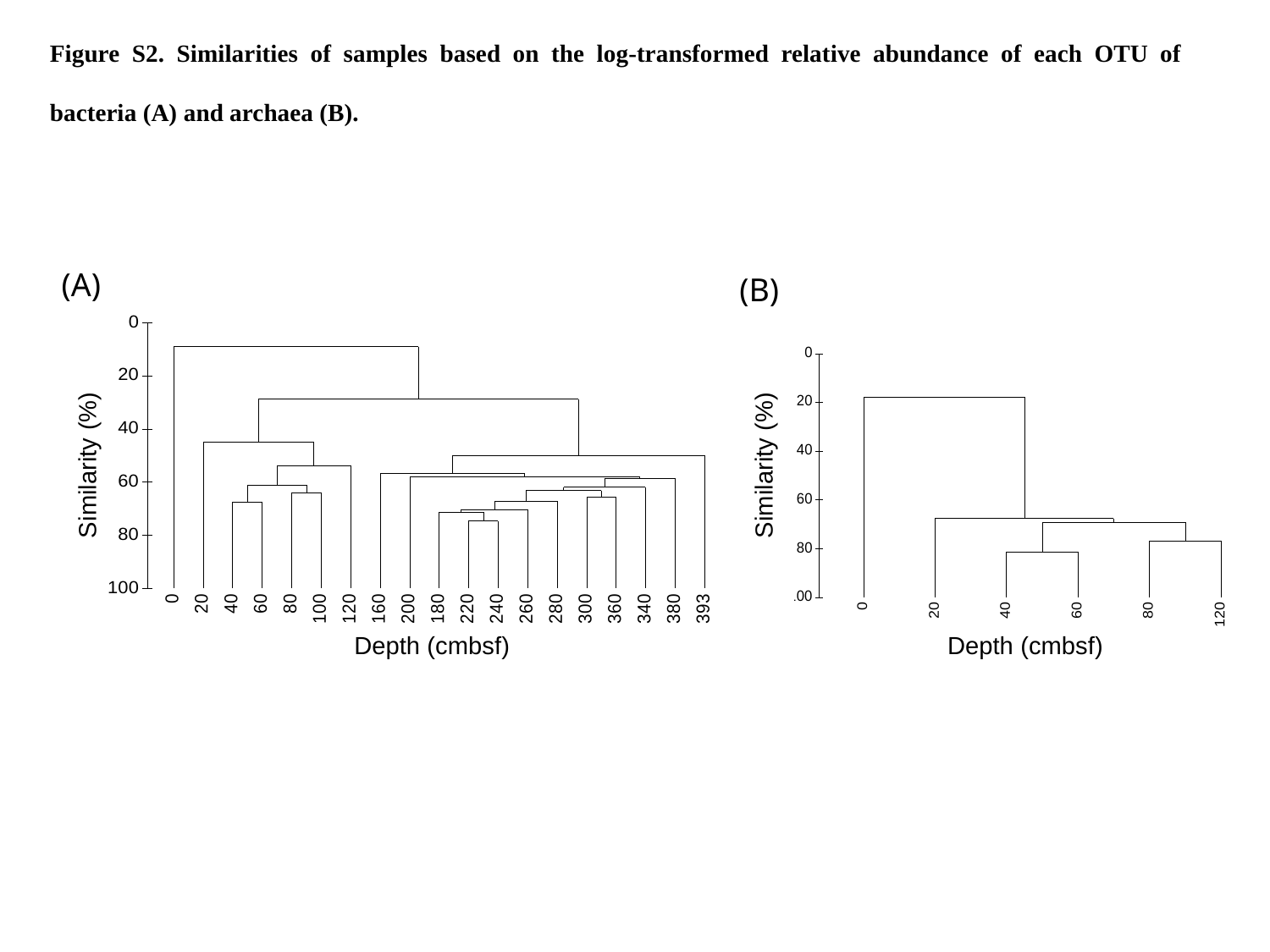

Figure S2. Similarities of samples based on the log-transformed relative abundance of each OTU of bacteria (A) and archaea (B).
(A)
(B)
Similarity (%)
Similarity (%)
Depth (cmbsf)
Depth (cmbsf)

## Slide 3
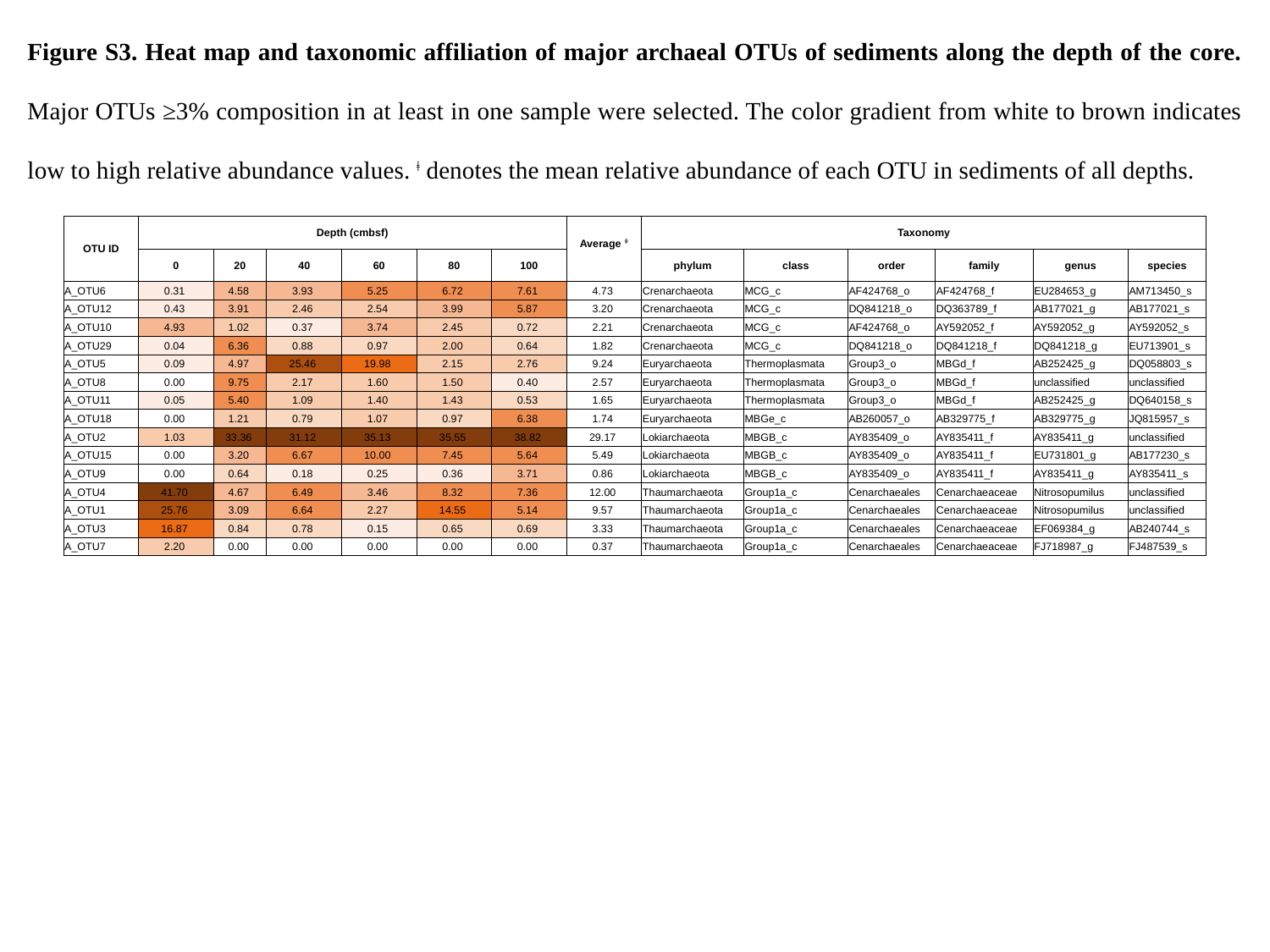

Figure S3. Heat map and taxonomic affiliation of major archaeal OTUs of sediments along the depth of the core. Major OTUs ≥3% composition in at least in one sample were selected. The color gradient from white to brown indicates low to high relative abundance values. ǂ denotes the mean relative abundance of each OTU in sediments of all depths.
| OTU ID | Depth (cmbsf) | | | | | | Average ǂ | Taxonomy | | | | | |
| --- | --- | --- | --- | --- | --- | --- | --- | --- | --- | --- | --- | --- | --- |
| | 0 | 20 | 40 | 60 | 80 | 100 | | phylum | class | order | family | genus | species |
| A\_OTU6 | 0.31 | 4.58 | 3.93 | 5.25 | 6.72 | 7.61 | 4.73 | Crenarchaeota | MCG\_c | AF424768\_o | AF424768\_f | EU284653\_g | AM713450\_s |
| A\_OTU12 | 0.43 | 3.91 | 2.46 | 2.54 | 3.99 | 5.87 | 3.20 | Crenarchaeota | MCG\_c | DQ841218\_o | DQ363789\_f | AB177021\_g | AB177021\_s |
| A\_OTU10 | 4.93 | 1.02 | 0.37 | 3.74 | 2.45 | 0.72 | 2.21 | Crenarchaeota | MCG\_c | AF424768\_o | AY592052\_f | AY592052\_g | AY592052\_s |
| A\_OTU29 | 0.04 | 6.36 | 0.88 | 0.97 | 2.00 | 0.64 | 1.82 | Crenarchaeota | MCG\_c | DQ841218\_o | DQ841218\_f | DQ841218\_g | EU713901\_s |
| A\_OTU5 | 0.09 | 4.97 | 25.46 | 19.98 | 2.15 | 2.76 | 9.24 | Euryarchaeota | Thermoplasmata | Group3\_o | MBGd\_f | AB252425\_g | DQ058803\_s |
| A\_OTU8 | 0.00 | 9.75 | 2.17 | 1.60 | 1.50 | 0.40 | 2.57 | Euryarchaeota | Thermoplasmata | Group3\_o | MBGd\_f | unclassified | unclassified |
| A\_OTU11 | 0.05 | 5.40 | 1.09 | 1.40 | 1.43 | 0.53 | 1.65 | Euryarchaeota | Thermoplasmata | Group3\_o | MBGd\_f | AB252425\_g | DQ640158\_s |
| A\_OTU18 | 0.00 | 1.21 | 0.79 | 1.07 | 0.97 | 6.38 | 1.74 | Euryarchaeota | MBGe\_c | AB260057\_o | AB329775\_f | AB329775\_g | JQ815957\_s |
| A\_OTU2 | 1.03 | 33.36 | 31.12 | 35.13 | 35.55 | 38.82 | 29.17 | Lokiarchaeota | MBGB\_c | AY835409\_o | AY835411\_f | AY835411\_g | unclassified |
| A\_OTU15 | 0.00 | 3.20 | 6.67 | 10.00 | 7.45 | 5.64 | 5.49 | Lokiarchaeota | MBGB\_c | AY835409\_o | AY835411\_f | EU731801\_g | AB177230\_s |
| A\_OTU9 | 0.00 | 0.64 | 0.18 | 0.25 | 0.36 | 3.71 | 0.86 | Lokiarchaeota | MBGB\_c | AY835409\_o | AY835411\_f | AY835411\_g | AY835411\_s |
| A\_OTU4 | 41.70 | 4.67 | 6.49 | 3.46 | 8.32 | 7.36 | 12.00 | Thaumarchaeota | Group1a\_c | Cenarchaeales | Cenarchaeaceae | Nitrosopumilus | unclassified |
| A\_OTU1 | 25.76 | 3.09 | 6.64 | 2.27 | 14.55 | 5.14 | 9.57 | Thaumarchaeota | Group1a\_c | Cenarchaeales | Cenarchaeaceae | Nitrosopumilus | unclassified |
| A\_OTU3 | 16.87 | 0.84 | 0.78 | 0.15 | 0.65 | 0.69 | 3.33 | Thaumarchaeota | Group1a\_c | Cenarchaeales | Cenarchaeaceae | EF069384\_g | AB240744\_s |
| A\_OTU7 | 2.20 | 0.00 | 0.00 | 0.00 | 0.00 | 0.00 | 0.37 | Thaumarchaeota | Group1a\_c | Cenarchaeales | Cenarchaeaceae | FJ718987\_g | FJ487539\_s |

## Slide 4
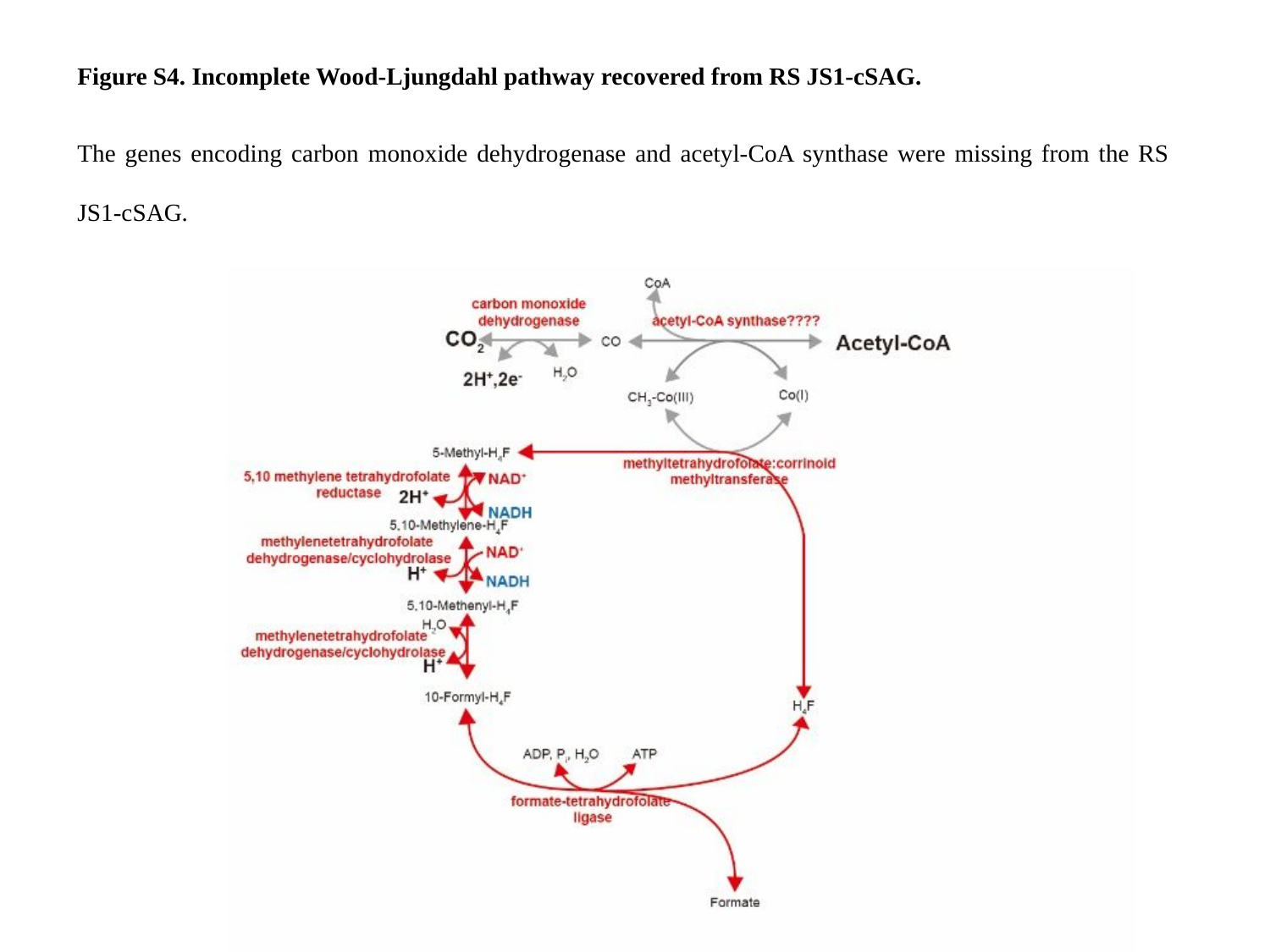

Figure S4. Incomplete Wood-Ljungdahl pathway recovered from RS JS1-cSAG.
The genes encoding carbon monoxide dehydrogenase and acetyl-CoA synthase were missing from the RS JS1-cSAG.
