# Supplementary material for: Genomic Insight Into the Predominance of Candidate Phylum Atribacteria JS1 Lineage in Marine Sediments
Source: Front Microbiol. 2018 Nov 29;9:2909. doi: 10.3389/fmicb.2018.02909 (PMC6281690; doi:10.3389/fmicb.2018.02909)
Supplement: Supplementary file 3 [file Data_Sheet_1.docx]

Supplementary file 1.16S rRNA sequences of major JS1 OTU (B_OTU1 and B_OTU3) and archaeal OTU, A­_OTU15 which showed a significant correlation with JS1 OTU (B_OTU1)

>B_OTU1 (Ca. Atribacteria)

GGATGAGCCTGCGTCCTATTAGTTAGTTGGTGGGGTAATGGCCTACCAAGACCACAATGGGTAGCCGGTCTGAGAGGATGTACGGCCACACTGGGACTGAGATACGGCCCAGACTCCTACGGGAGGCAGCAGTGGGGAATATTGCGCAATGGGGGAAACCCTGACGCAGCGACGCCGCGTGGATGATGAAGGCCCTTGGGTTGTAAAATCCTGTTCTGGGGGAAGAAAGCTTAAAGGTCCAATAAACCCTTAAGCCTGACGGTACCCCA-AGAGAAAGCTCCGGCT-AATTATGTGC

>B_OTU3 (Ca. Atribacteria)

GGATGAGCCTGCGTCCTATTAGTTAGTTGGTGGGGTAATGGCCTACCAAGACCACAATGGGTAGCCGGTCTGAGAGGATGTACGGCCACACTGGGACTGAGATACGGCCCAGACTCCTACGGGAGGCAGCAGTGGGGAATATTGCGCAATGGGGGAAACCCTGACGCAGCGACGCCGCGTGGATGATGAAGGCCTTTGGGTTGTAAAATCCTGTTCTGGGGGAAGAAAGCTTAAGGGTCCAATAAACCTTTAAGTTTGACGGTACCCCA-AGAGAAAGCTCCGGCT-AATTATGTGC

> A_OTU15 (Lokiarchaeota)

GGCTCACCAAGCCGATAATCGATAGGGGCCGTGAGAGCGGGAGCCCCAAGATGGGTACTGAGACAGCGACCCAGGCCTTACGAGGCGCAGCAGGTACGAAACCTCCGCAATACACGAAAGTGTGACGGGGTTACCCAAAGTGTTCTTATAGAACTGTGGCAGGTGAGTAATGTCCCCTGCTAGAAAGGAGAGGGCAAGGCTGGTGC
